# Sociodemographic characteristics of women who died by suicide in India from 2014 to 2020: findings from surveillance data

**DOI:** 10.1016/S2468-2667(23)00028-2

**Published:** 2023-04-27

**Authors:** Rakhi Dandona, Sibin George, G Anil Kumar

**Affiliations:** a Public Health Foundation of India, Gurugram, India; b Institute for Health Metrics and Evaluation, University of Washington, Seattle, WA, USA

## Abstract

**Background:**

Women in India have twice the suicide death rate (SDR) compared with the global average for women. The aim of this study is to present a systematic understanding of sociodemographic risk factors, reasons for suicide deaths, and methods of suicide among women in India at the state level over time.

**Methods:**

Administrative data on suicide deaths among women by education level, marital status, and occupation, and reason for and method of suicide were extracted from the National Crimes Record Bureau reports for years 2014 to 2020. We extrapolated SDR at the population level for Indian women by education, marital status, and occupation to understand the sociodemography of these suicide deaths for India and its states. We reported the reasons for and methods of suicide deaths among Indian women at the state level over this period.

**Findings:**

SDR was higher among women with education of class 6 or more (10·2; 95% CI 10·1–10·4) than those with no education (3·8; 3·7–3·9) or education until class 5 (5·4; 5·2–5·5) in India in 2020, with similar patterns in most states. SDR declined between 2014 and 2020 for women with education until class 5. Women currently married accounted for 28 085 (63·1%) of 44 498 suicide deaths in India, 8336 (56·2%) of 14 840 in less developed states, and 19 661 (66·9%) of 29 407 in more developed states in 2020. For India, women currently married had a significantly higher SDR (8·1; 8·0–8·2) than those never married in 2014. However, women who never married had a significantly higher SDR (8·4; 8·2–8·5) in 2020 than those who were currently married. Many individual states in 2020 had similar SDR for women who never married and those who are currently married. Housewife as an occupation accounted for 50% or more of suicide deaths from 2014 to 2020 in India and its states. Family problems was the most common reason for suicide from 2014 to 2020, accounting for 16 140 (36·3%) of 44 498 suicide deaths in India, 5268 (35·5%) of 14 840 in less developed states, and 10 803 (36·7%) of 29 407 in more developed states in 2020. Hanging was the leading mean of suicide from 2014 to 2020. Insecticide or poison consumption was the second leading cause of suicide, accounting for 2228 (15·0%) of all 14 840 suicide deaths in less developed states and 5753 (19·6%) of 29 407 in more developed states, with a near 70·0% increase in the use of this method from 2014 to 2020.

**Interpretation:**

The higher SDR among women who have received an education, similar SDR between women currently married and never married, and variations in the reasons for and means of suicide at the state level highlight the need to incorporate sociological insights into how the external social environment can matter for women to better understand the complexity of suicide and determine how to effectively intervene.

**Funding:**

Bill & Melinda Gates Foundation.

## Introduction

Several calls have been made for a national suicide prevention strategy to address the prevailing sociodemographic factors and other risk factors to reduce deaths by suicide in India.^1^, ^2^, ^3^, ^4^ As the Indian government continues to consider these calls, an important prerequisite for effective suicide prevention is to develop strategies that can address the risks or means of suicide among vulnerable people. For example, the Brazilian conditional cash-transfer programme could reduce suicide in Brazilian municipalities by mitigating the effect of poverty on suicide incidence,^5^ and a systematic review from 16 countries highlighted that national bans on highly hazardous pesticides are effective in reducing pesticide-specific suicide rates and overall suicide rates.^6^

Indian women are a vulnerable group for suicide deaths. The Global Burden of Disease (GBD) Study documented that Indian women had twice the suicide death rate (SDR) compared with the global average for women in 2016.^3^ This pattern continued in 2019 with SDR for Indian women estimated at 12·7 and the global average at 6·1 per 100 000 women.^7^ Importantly, suicide was the leading cause of death in the 15–39 year age group, with 71·2% of the suicide deaths among women occurring in this age group in India.^3^ The GBD study did not provide data on suicide deaths by education level, marital status, or occupation of women, nor on risk factors for and means of suicide deaths at the individual level, which are imperative for planning targeted interventions.^3^ In this context, we undertook a detailed analysis of deaths by suicide among women in India for the years 2014 to 2020 available from the National Crime Records Bureau (NCRB)^8^ with the aim of exploring the sociodemographic factors and other risk factors for women that could facilitate identification of specificity in interventions to reduce suicide deaths among women. Despite the known issues of under-reporting and inadequate reporting in the NCRB,^9^, ^10^, ^11^ it remains the only source that provides data on the reasons for suicide and means of suicide at a large scale that can facilitate prioritisation of immediate actions for suicide prevention. Furthermore, we extrapolated SDR at population level for Indian women by education and marital status to understand the sociodemography of these suicide deaths. Lastly, we highlighted research and data priorities with direct relevance to the development and implementation of population-level strategies to reduce suicide deaths among women in India.

**Research in context**

**Evidence before this study**

As estimated by the Global Burden of Disease (GBD) Study 2019, Indian women have twice the suicide death rate (SDR) compared with the global average for women. The GBD Study does not provide SDR by sociodemographic risk factors other than age for India and its states over time. We searched PubMed and publicly-available reports for estimates of SDR across the states of India using the search terms “suicide”, death”, “India”, “women”, “self-harm”, “state-level”, “education”, “marital status”, “occupation”, “reason”, and “method”, on June 1, 2022, without language or publication date restrictions. We predominately found publications from the GBD Study by region and age, publications using National Crimes Record Bureau (NCRB) data until 2012 on occupation, reasons, and methods for suicide deaths, publications on domestic violence and its association with suicide, and publications on suicidal behaviour from patient and population assessments. No studies that comprehensively described suicide death trends in every state of India by education and marital status were found.

**Added value of this study**

Our study provides a comprehensive assessment of SDR by education status and marital status from 2014 to 2020 for each state of India, distribution of occupation of deceased women, and reasons for and methods of suicide among Indian women. Women with education of class 6 or more have a higher SDR in most states than those with education of class 5 or higher or no education, with variation seen in some states, where women with no education had the highest SDR. Although the distribution of marital status in the NCRB data suggests a higher proportion of women who were currently married among those who died by suicide, the SDRs suggest a similar burden for women currently married and never married women, with a higher SDR for women who never married in 2020. Family problems dominated the reasons for these suicide deaths followed by health and marriage. Hanging was the predominant method of suicide followed by consumption of insecticides or poison.

**Implications of all the available evidence**

Quantitative research of administrative data can inform the epidemiology of suicide deaths; however, the findings of this report support the importance of culture in understanding suicidal behaviour to prevent these deaths. Cross-state studies with robust sociological methods that can add depth to contextualising the determinants of suicidal behaviour among Indian women to improve suicide prevention planning along with research to facilitate better decision making are urgently needed.

## Methods

### Data from the NCRB

The primary source of NCRB data is the First Information Report (FIR) completed by a police officer for suicide death, which is compiled at the state level and then provided to the NCRB for compilation. FIR is a document prepared by the police when they receive information about occurrence of a cognisable offence either by the victim of the cognisable offence or by someone on behalf of the victim.^12^, ^13^ It captures the date, time and location of offence, details of the offence, details of victim and person reporting the offence, and steps taken by the police after receiving these details. The NCRB reports provide summary data on deaths by suicide on the basis of these FIRs in the public domain in a predefined format, which we used from 2014 to 2020 for this analysis. The details of data extracted and used are described hereafter.

The NCRB reports provide the number of deaths by suicide each year, and distribution of the victims by educational status, marital status, and occupation in predefined tabulated categories for India and all its states.^8^ The NCRB reports do not provide individual level data. We extracted these data for women. The NCRB changed the reporting categories for educational status, marital status, and occupation from 2014 onwards, hence we considered data only for years 2014 to 2020 to ensure that standardisation across the reporting categories was maintained. Data on suicide deaths in predefined age categories were available only for India and not for individual states, and were extracted for years 2014 to 2020. We also extracted data on the means of suicide death and the reason for suicide in women in India and its states for years 2014 to 2020. We combined some categories for these variables for a meaningful interpretation by reducing the redundancy of the original smaller groupings (appendix 1 pp 3–4).

### Data from the National Sample Survey (NSS)

Under the backing of the Ministry of Statistics and Programme Implementation, the NSS Organisation has been undertaking the NSS every year since 1950 with the aim of providing comprehensive and continuing information on social, economic, and demographic conditions in the country.^14^ We extracted data on distribution of education and marital status for women aged 10 years or more from NSS rounds 71 and 75, which corresponded to years 2014 and 2017–18, respectively, and data on occupation that were available only for NSS round 75 (year 2020).^15^, ^16^ Round 75 was the latest round available for this survey. We recategorised the NSS data to make these comparable with the NCRB categories for education, marital status, and occupation (appendix 1 p 5).

### Data analysis

We estimated the SDR per 100 000 women for each year from 2014 to 2020 for India and its states. For this estimation, we considered the number of deaths by suicide for women reported in the NCRB and used the GBD study state-wise annual population estimates for women aged 10 years or older as the denominator for each year.^17^ We extrapolated the denominator for year 2020 from 2019 using linear projection of the population growth rate from 2018 to 2019. Because the lowest age category available in the NCRB was 10–17 years, we estimated SDR for women aged 10 years or older. The age-specific SDR was estimated only for India for the years 2014 to 2020 on the basis of data availability in the NCRB.

Furthermore, we estimated SDR for women by education and marital status for the years 2014 and 2020, and by occupation for year 2020. To estimate the denominator by education, marital status, and occupation categories, we applied the proportional distribution of women aged 10 years or older by education, marital status, and occupation categories from the NSS to the estimated women population aged 10 years or older, as per the GBD study.^18^ Using this estimated denominator, we calculated the SDR as follows: for women with no education, those who had studied class 1–5, class 6–8, class 9–12, and those who had graduate level or higher education; for women never married, currently married, and previously married; and for women who were housewives, professionals, self-employed, had their wages paid daily, students, and unemployed, among other categories.

All SDR are reported per 100 000 women aged 10 years or older with 95% CIs estimated for India, individual states, and by states grouped by development status in Microsoft Excel.^19^, ^20^ The states were grouped as less developed or more developed.^21^ The category that was less developed included the states of Bihar, Madhya Pradesh, Jharkhand, Rajasthan, Uttar Pradesh, Chhattisgarh, Odisha, Assam, and seven northeastern states (Arunachal Pradesh, Assam, Manipur, Meghalaya, Mizoram, Nagaland, Sikkim, and Tripura).^21^, ^22^, ^23^ The remaining states were categorised as more developed. We reported findings for the undivided state of Jammu and Kashmir.

We reported the trends in the reasons for suicide and the means for suicide by marital status and occupation from the NCRB to understand whether the reasons or means changed over time across India or states from years 2014 to 2020. All analyses were run in Microsoft Excel 2010 and R version 4.1.2 (tidyverse, readxl, and ggplot2). No ethics approval was required for this analysis as the data from the NCRB, NSS, and GBD study were available in the public domain. All raw data are available in appendix 2.

### Role of the funding source

The funder of the study had no role in data collection, data analysis, data interpretation, writing of the manuscript, or the decision to submit for publication.

## Results

As per NCRB reports, the number of deaths by suicide for women increased marginally from 42 521 in 2014 to 44 498 in 2020 in India, an increase of 4·6%. The SDR for women in India decreased by 4·5% (95% CI –5·8 to –3·2) from 8·2 (8·1 to 8·3) in 2014 to 7·8 (7·7 to 7·9) per 100 000 women in 2020 (appendix 1 pp 6–7). The SDR was consistently higher in the more developed states than the less developed states, with it being 2·15 times (95% CI 2·11 to 2·19; rate-ratio test p<0·0001) higher in 2020 in the more developed states. The less developed states showed an increase of 5·6% (3·2 to 8·1) in SDR from 4·6 (4·5 to 4·6) in 2014 to 4·8 (4·7 to 4·9) per 100 000 women in 2020, which was statistically significant (Z test p<0·0001). The SDR in more developed states reduced significantly (Z test p<0·0001) from 2014 to 2020 (appendix 1 pp 6–7).

We reported the distribution of education status as reported in the NCRB among the women who died by suicide for 2014 and 2020 for India and its states (appendix 1 pp 8–9). Women with an education level of class 9–12 accounted for the highest proportion of female suicide deaths in India in 2014 (12 658 [29·8%] of 42 521) and in 2020 (17 046 [38·3%] of 44 498); however, this pattern varied significantly by individual state (appendix 1 pp 8–9).

**Figure 1:**
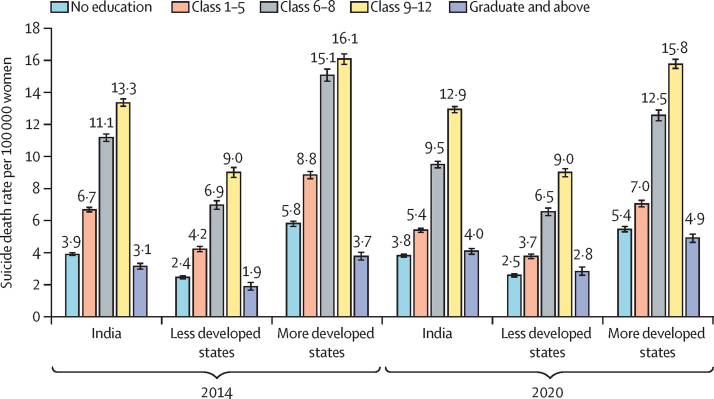
Comparison of suicide death rate per 100 000 women in 2014 and 2020 by level of education for India and grouping of states by development status Error bars denote 95% CIs.

For India in 2020, the SDR among women with education to class 6–8 (9·5; 95% CI 9·3–9·7) and in those with education to class 9–12 (12·9; 12·7–13·1) was higher than those with no education (3·8; 3·7–3·9) or education class 1–5 (5·4; 5·2–5·5). A similar pattern was seen in most states in 2020, with some variation seen in this pattern for Chhattisgarh, Madhya Pradesh, Meghalaya, Rajasthan, Uttar Pradesh, Andhra Pradesh, Haryana, Punjab, and Telangana, wherein the SDR in women with no education was also high (appendix 1 p 10). The SDR among women by education showed an interesting pattern between 2014 and 2020 with significant narrowing of SDR between those with education level to class 6–8 and class 9–12 because of a decrease in SDR in those with education to class 6–8 (–15·0%; 95% CI –17·8 to –12·3) during this period. The decrease in SDR in women with education to class 6–8 was higher in the more developed states (–16·7%; –20·1 to –13·4) than less developed states (–6·1%; –11·2 to –1·0). A decline in SDR was also seen in 2020 among women with education to class 1–5, again with a higher decline in the more developed states (–20·3%; –23·7 to –16·8) than the less developed states (–11·0%; –16·2 to –5·8; appendix 1 pp 10–11; figure 1). A more or less stable pattern was seen for SDR among women with no education in the less developed states, but a statistically significant decline was seen in the more developed states over this period (–5·8%; –10·0 to –1·6, p=0·0065).

We reported the distribution of marital status among the women who died by suicide as reported in the NCRB for years 2014 to 2020 (appendix 1 pp 12–13). Women who were currently married accounted for 28 085 (63·1%) of 44 498 suicide deaths among women in India, 8336 (56·2%) of 14 840 in the less developed states, and 19 661 (66·9%) of 29 407 in the more developed states in 2020. However, women who never married accounted for 11 826 (26·6%) of 44 498 suicide deaths in India, 4552 (30·7%) of 14 840 in less developed states, and 7214 (24·5%) of 29 407 in more developed states.

**Figure 2:**
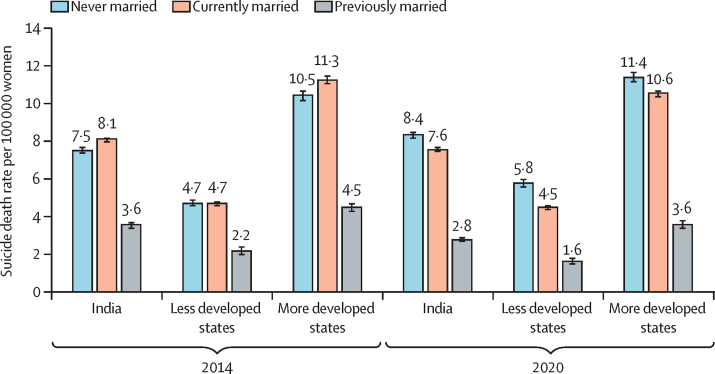
Suicide death rate per 100 000 women by marital status for India and grouping of states by development status, 2014 and 2020 Error bars denote 95% CIs.

**Figure 3:**
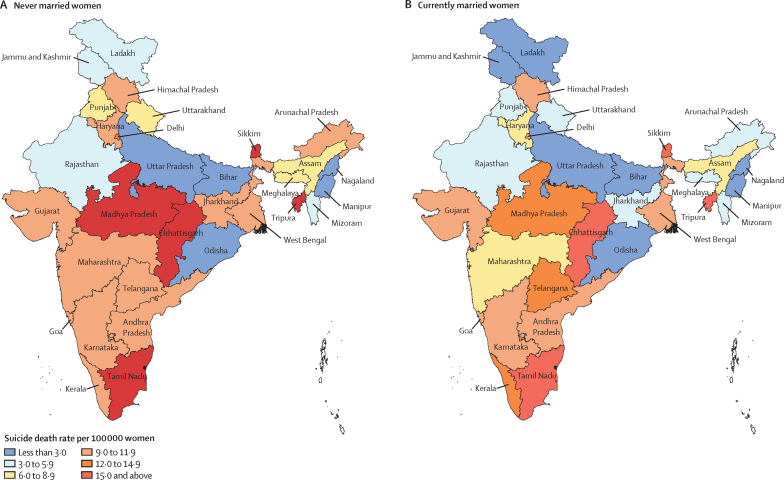
Suicide death rate per 100 000 women for women never married and currently married for the states of India, 2020

However, SDR at the population level for India on the basis of marital status changed significantly from 2014 to 2020, irrespective of the development status of the state (figure 2; appendix 1 p 14). For India, women who were currently married had a significantly higher SDR (8·1; 95% CI 8·0–8·2) than women who never married in 2014, but women who never married had a significantly higher SDR (8·4; 8·2–8·5) in 2020 than those who were currently married (figure 2; appendix 1 pp 10–11). Many individual states in 2020 had similar SDRs for the women who never married and those who were currently married, with some variation in a few states such as Arunachal Pradesh, Chhattisgarh, Kerala, Madhya Pradesh, Maharashtra, Punjab, and Telangana (figure 3; appendix 1 p 10).

**Figure 4:**
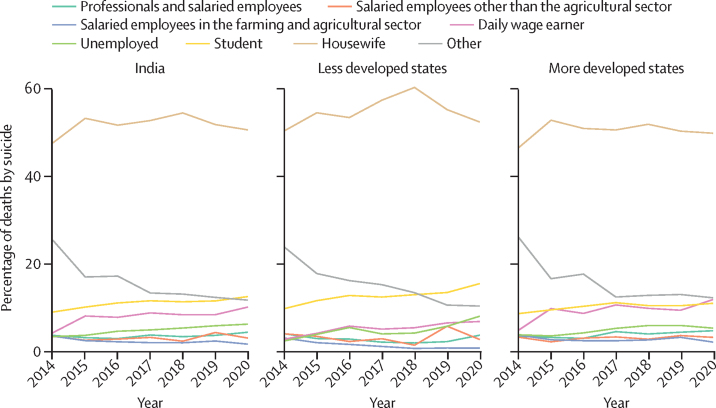
Occupation of women who died by suicide in India and by grouping of states by development status, 2014 to 2020

Women who were housewives or who had house duties as an occupation accounted for 50% or more of the suicide deaths from 2014 to 2020 in India, and in the less developed and more developed states (figure 4). The distribution of remaining occupation categories did not change much from 2014 to 2020, except for the category recorded as others, which documented a sharp decline in both the less developed and more developed states. A similar proportion of suicide deaths were recorded with occupation as student or daily wage earner in the more developed states, whereas suicide deaths with occupation as student were recorded in a higher proportion than daily wage earner in less developed states in 2020.

SDR by occupation was estimated only for year 2020 (appendix 1 p 15). SDR was highest among women who were unemployed followed by women who were employed in any kind of employment, compared with housewives and students. SDR was significantly higher among daily wage earners and self-employed women in more developed states than in less developed states, whereas SDR in less developed states was significantly higher than in more developed states for women who were professionals or salaried employees.

The age-specific SDR for India showed a stable pattern from 2014 to 2020 for all age groups, except in women aged 60 years or older, which showed a decline during this period (appendix 1 p 16). The highest age-specific SDR during this period was seen for the 45–59 year age group, ranging from 21·1 (95% CI 20·8–21·4) in 2014 to 18·5 (18·3–18·8) per 100 000 women in 2020.

**Figure 5:**
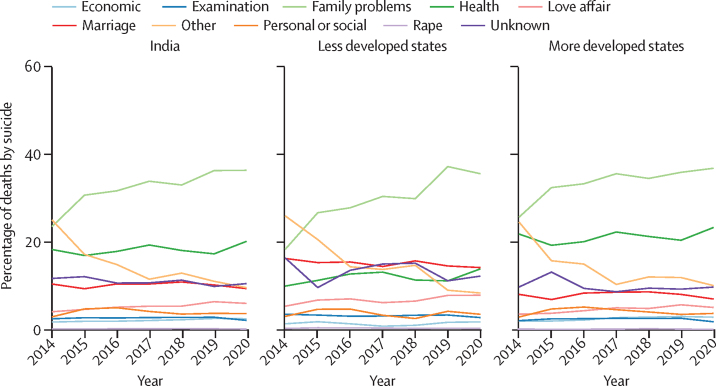
Reasons for deaths by suicide among women from 2014 to 2020 for India and grouping of states by development status

Family problems were reported as the most common reason for suicide from 2014 to 2020, with an increasing trend seen for this reason over these years for India and the less developed and more developed states (figure 5). Family problems accounted for 16 140 (36·3%) of 44 498 suicide deaths in India, 5268 (35·5%) of 14 840 in less developed states, and 10 803 (36·7%) of 29 407 in more developed states in 2020. Reporting of other reasons decreased substantially over the same period. Health was the second-highest recorded reason for suicide in India (mean 18%) and in the more developed states (mean 21%), whereas marriage was recorded as a reason more frequently in less developed states (mean 15%). Reason for suicide being unknown was the fourth leading reason reported for suicide deaths irrespective of the development status of states.

**Figure 6:**
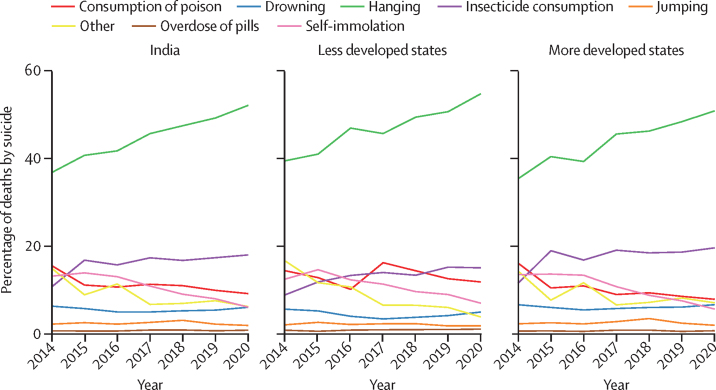
Means of deaths by suicide among women from 2014 to 2020 for India and grouping of states by development status

Hanging was increasingly the leading mean of suicide deaths among women from 2014 to 2020 for India as a whole and for less developed and more developed states (figure 6).

From 2014 to 2020, the proportion of deaths caused by hanging increased by 38·7% (95% CI 35·7–41·7) in less developed states and 43·8% (41·6–46·1) in more developed states from 2014 to 2020 (z test p<0·0001). Insecticide or poison consumption was the second leading mean of suicide, accounting for 2228 (15·0%) of all 14 840 suicide deaths in less developed states and 5753 (19·6%) of all 29 407 suicide deaths in more developed states in 2020, with a similar increase in this method of suicide from 2014 to 2020 (69·1%, 95% CI 60·5–77·6 in less developed states and 70·6%, 65·5–75·7 in more developed states; Z test p<0·0001). Self-immolation decreased over this period and accounted for less than 10% of suicides in both types of states (Z test p<0·0001).

Hanging was the leading mean of suicide in all the states except Odisha, Himachal Pradesh, and Jammu and Kashmir, where it was insecticide or other poison consumption (appendix 1 pp 17–18). In Telangana, hanging was the leading mean of suicide between 2014 and 2018, which was overtaken by insecticide or other poison consumption in 2019 and 2020 (appendix 1 pp 17–18).

## Discussion

To our knowledge, this is the first comprehensive assessment of suicide deaths among Indian women offering insights into sociodemographic factors spanning over a period of 7 years. The predominance of suicide deaths among women who have received an education and similar SDR between those currently married and never married at the population level, and variations in the reasons for and means of suicide at the state level highlight the need for specific and multisectoral interventions to address these deaths. Importantly, the findings of this report support the importance of incorporating sociological insights into how the external social environment can matter to suicide and suicide prevention among women, which might help us better understand the complexity of suicide and determine how to effectively intervene.

Our analysis is limited by the limitations of the NCRB data, which depend on the availability and quality of data recorded at the local level by the police,^2^, ^3^, ^24^ and hence the findings of this report should be interpreted within this limitation. The NCRB data are not used in the GBD study SDR estimates.^3^ A comparison of SDR estimates between the two sources has already highlighted an under-reporting by 50% of deaths by suicide among women in the NCRB compared with the GBD study, with variations at the state level.^25^ Another limitation is that the NCRB data are available in pretabulated fixed formats and not at the individual level, which limits the extent of disaggregated analysis. However, despite these limitations, the NCRB remains the only administrative data source to understand sociodemographic factors and other risk factors over time and by state for all types of injuries, including suicides. It is not fully clear whether the COVID-19 pandemic might have affected data collection for a few months in 2020; however, the pattern presented here from 2014 to 2020 does not suggest any major bias in the data for 2020. Although the NCRB data showed that SDR is higher in the more developed states than in the less developed states, as in the GBD study,^3^ the pattern by sociodemographic profile and reasons for and means of suicide deaths were similar in both types of states. Therefore, we focused this discussion on the insights from sociodemographic factors and other risk factors for suicide deaths among women according to the NCRB that could facilitate interventions to address suicide deaths among women.

The framework for action for implementation of Sustainable Development Goal 4 aims to transform the lives of women and men through education, recognising the important role of education as a main driver of development and for achieving the other proposed Sustainable Development Goals.^26^ The finding of a higher SDR among women with education class 6 and higher in most states raises concerns on the extent of empowerment that education provides to Indian women, given that education is considered one of the most powerful tools for female empowerment, allowing women to confront their traditional role, respond to challenges, and change their positions in society.^27^ An analysis of NCRB data from 2001 to 2011 with the assumption that lower total fertility rate corresponded to greater female autonomy suggested that at constant levels of economic development, lower total fertility rate might reduce suicide risk among women.^28^ Much of the literature on the education and health of women from developing countries is about their impact on child health, with limited understanding of women's education in relation to suicide deaths, suicidality, or their mental health in general.^29^, ^30^, ^31^ However, that a higher SDR was estimated for women with no education in some Indian states in this analysis highlights the paucity in our understanding of education as either a risk factor or protective factor for suicide among women. Higher suicidality in women with lower educational attainment has also been reported in the National Mental Health Survey of India 2015–16.^1^

This paucity in our understanding of the sociodemography of suicide deaths in women is further highlighted by the data on SDR by marital status. The distribution of women who died by suicide in the NCRB data shows a predominance of women who were currently married; however, the SDR at the population level was higher for the women who were currently married in 2014 and for those who were never married in 2020. The different SDR pattern at the population level and the change in patterns between 2014 and 2020 was unexpected. The GBD study estimated one of the highest SDRs among younger women in the 15–29 year age group.^3^ The higher under-reporting of suicide deaths in younger women than in women who were older has been suggested to be a result of stigma and shame associated with these deaths in India.^25^ Much of the literature on suicidality and marital status of women is focused on intimate partner violence in women who are currently married, suggesting associations of an increased risk for depression and suicide.^32^, ^33^ There is also a suggestion that economic empowerment in women is a risk for intimate partner violence and that working for cash increases a woman's risk in countries where few women work.^34^, ^35^, ^36^ The SDR by occupation in the year 2020 showed a higher SDR for women who worked outside the home when compared with housewives, with the highest SDR for those who were unemployed. Although a significant association between depression and suicide death was reported in India for women,^37^, ^38^ there was not much understanding of how both economic empowerment and intimate partner violence translated into suicide deaths for women who were currently married. Our understanding of women who were never married in India, and why SDR in these women increased over time, is even more limited. High suicide deaths in adolescent girls has gained attention with suicides having surpassed maternal mortality as the leading cause of death globally in this population group, and a higher risk of depression in addition to early marriage are cited as possible reasons for this high suicide death rate.^39^, ^40^, ^41^ However, the reasons for suicide available as pretabulated NCRB formats in the public domain did not allow us to deeping our understanding of these issues. If one were to make assumptions about the possible reasons for suicide among women who never married from the available data, these assumptions could include study-related examinations, love affairs, family problems, or personal and social problems. Similarly, one could assume that the occupation recorded as student in the NCRB data could be applicable predominately to women who never married.

There was a change in the pattern of reasons for suicide from 2014 to 2020, with an increase in family problems as a cause and decrease in other reasons for suicide, and reasons also varied according to the level of state development. Nearly 20% of reasons were accounted for by unknown reasons and other reasons combined in 2020, offering limited understanding for the purpose of prevention. The reasons provided in the tabulated formats in the NCRB data were not nuanced enough to offer insights that could facilitate specificity in interventions by state, age, marital status, or education. In analyses of NCRB data on domestic violence and sexual violence,^41^, ^43^ we demonstrated the limitations in these data in not only understanding patterns in these crimes over time, but also in understanding issues with the documentation and recording of crimes by police. Hence, more insights are needed into how the reasons for suicide deaths are recorded by the police and whether there are any influences on recording. For example, it is possible that some police personnel record marriage as a specific reason for suicide, whereas some might record it as family problem. Even though NCRB data are a passive surveillance source for suicide deaths, efforts can be made to improve the quality of information collected by the police during their routine tasks to improve use of these data for planning action.^42^, ^43^

Hanging continues to be the most commonly used method of suicide in India for all sexes.^2^, ^3^, ^44^ It is important to note that nearly one in four suicide deaths in women was still caused by consumption of insecticide or poison irrespective of the type of state, and this method was the leading methods of suicide in Telangana. On the one hand, there is evidence from the NCRB data of a decline in the use of insecticide or poison over time,^2^, ^44^ and on the other hand, a systematic review estimated that there could be nearly 50 000 or more pesticide-related suicide deaths every year, higher than that reported in the NCRB data.^45^ India has banned the production, distribution, and sale of 18 pesticides in 2018, and it is probable that this ban will be expanded to an additional 27 toxic pesticides.^46^ With nearly 25% of the suicide deaths among women still caused by the consumption of insecticide or poison, further measures are needed to address this method of suicide in India. Importantly, that hanging is the predominant method of suicide, access to which is difficult to restrict, indicates the need to understand the context in which hanging occurs and who dies by hanging.

The findings from this analysis corroborate a study that highlighted that the relatively high SDR among women in low-income and middle-income countries compared with high-income countries might be related to the higher level of institutional discrimination women experience in these countries, including women having restricted access to productive and financial assets and to justice, or unequal rights with regard to citizenship, household responsibilities, divorce, and inheritance.^47^ Quantitative research of administrative data such as this analysis can inform the epidemiology of suicide deaths; however, the findings of this report support the importance of culture in understanding suicidal behaviour to prevent these deaths.^48^, ^49^ Durkheimian insight into why people die by suicide (which states that absence of meaningful social relationships, which support us during difficult times and celebrate us when times are good, is extremely harmful to individual wellbeing) has been one of the best contributions of sociology; the empirical and theoretical advances over the past years has shown that incorporating sociological insights into how the external social environment can matter to suicide and suicide prevention could help us better understand the complexity of suicide and determine how to effectively intervene.^49^, ^50^ However, there is paucity of such studies.^51^ Cross-state studies that can add depth to contextualising the determinants of suicidal behaviour among Indian women and studies that can assist with causal inference to improve evidence-based suicide prevention planning and facilitate policy development and assessment of real-world impact of interventions on suicide deaths are urgently needed.^52^ Furthermore, studies that compare women who live in different states but who share a cultural background can elucidate how culture influences suicidal behaviour compared with various other aspects of suicidal behaviour. This aspect is important, given that suicide death rates have been found to be more strongly correlated with shared cultures, languages, and religion than shared geographic boundaries and proximity in a multicentre study on suicidal behaviour.^53^ Importantly, working with women with lived experience can help us understand how context and culture influence suicidal behaviour.^51^

In conclusion, the NCRB data do allow for some insights into suicide deaths in women in India. However, these insights are not necessarily the same as those offered by other data sources, which are also limited in explaining risk factors for suicide deaths. Qualitative research grounded in robust sociological methods is urgently needed for a deeper and more contextualised understanding of suicide deaths along with research to facilitate better decision making to prevent these deaths among Indian women.

## Data sharing

The suicide death data used in these analyses are available at the NCRB website (https://ncrb.gov.in) and from the corresponding author on request. The GBD population data used in these analyses are available at GBD Results Tool GHDx (https://healthdata.org). The NSS data are available from http://www.icssrdataservice.in/datarepository/index.php/catalog/107 and http://microdata.gov.in/nada43/index.php/catalog/152.

## Declaration of interests

The authors declare that they have no competing interests.
