# Supplementary material for: Sociodemographic characteristics of women who died by suicide in India from 2014 to 2020: findings from surveillance data
Source: Lancet Public Health. 2023 Apr 27;8(5):e347–55. doi: 10.1016/S2468-2667(23)00028-2 (PMC10165469; doi:10.1016/S2468-2667(23)00028-2)
Supplement: Supplementary appendix [file mmc1.pdf]

THE LANCET
Public Health

Supplementary appendix 1

This appendix formed part of the original submission and has been peer reviewed. We post it as supplied by the authors.

Supplement to: Dandona R, George S, Kumar GA. Sociodemographic characteristics of women who died by suicide in India from 2014 to 2020: findings from surveillance data. *Lancet Public Health* 2023; **8:** e347–55.

16 March 2023

Revision

Socio-demographic characteristics of women who died by suicide in India from 2014 to 2020: findings from administrative data

Rakhi Dandona, Sibin George, G. Anil Kumar

Web Appendix

Correspondence to: Prof. Rakhi Dandona, rakhi.dandona@phfi.org

Table of contents

1: Re-categorisation of the National Crimes Record Bureau (NCRB) categories of education, marital status, occupation, reasons and means of suicide deaths for analysis. --------------------------------------- 3

2: Re-categorisation of the National Sample Survey (NSSO) categories of education, marital status and occupation for analysis.--- 5

3: Suicide death rate per 100,000 women for India and grouping of states by development status, 2014 to 2020.* Error bars denote 95% confidence interval. -- 6

4: Suicide death rate per 100,000 women in India and states, 2014 to 2020.* ------------------------------ 7

5. Distribution of deaths by suicide of women by their education level for Indian states as reported by the National Crimes Report Bureau, 2014 and 2020. -- 8

6. Suicide death rate per 100,000 women for India and its states - overall, by level of education and marital status, 2020. --10

7. Suicide death rate per 100,000 women for India and its states-overall, by level of education and marital status in the year 2014. ---11

8. Distribution of marital status of women who died by suicide from 2014 to 2020 for India and its states as reported in the National Crimes Record Bureau Report. NM denotes never married, CM currently married, PM previously married, and OTH other. --12

9. Suicide death rate per 100,000 women for never married and currently married women for the states of India, 2014.* --14

10. Suicide death rate per 100,000 women for India and state categories by occupation, 2020.* ------15

11. Age-specific suicide death rate per 100,000 women for India, 2014 to 2020.* ----------------------16

12. Distribution of means of suicide death among women from 2014 to 2020 for India and its states as reported in the National Crimes Record Bureau Report. HG denotes hanging, IC insecticide consumption, SI self-immolation, CP consumption of poison, OTH other, DR drowning, JP jumping, and OP overdose of pills. ---17

1: Re-categorisation of the National Crimes Record Bureau (NCRB) categories of education, marital status, occupation, reasons and means of suicide deaths for analysis.

	NCRB category	Re-categorisation
Education	No education	No education
	Primary (up to class–5th)	Class 1-5
	Middle (up to class–8th)	Class 6-8
	Matriculate/Secondary (upto class 10th)	Class 9 -12
	Higher Secondary/ Intermediate/ Pre-University (up to class–12th), Diploma/Certificate/ ITI	
	Graduate and above: Professionals (MBA etc.)	Graduate and above
	Status not known	Status not known
Marital status	Unmarried	Never married
	Married	Currently married
	Widowed/Widower, Divorcee, Separated	Previously married
	Others, Status not known	Others
Occupation	Government Servants (Central/UT Govt. Servants), Government Servants (State Govt. Servants), Government Servants (Other Statutory Body/etc.), Private sector undertaking: Public sector undertaking	Professionals/Salaried employee
	Housewife	Housewife
	Self-employed Persons [Business(Vendor)], Self-employed Persons [Business(Tradesmen)], Self-employed Persons [Business(Other Business)], Other Self-employed Persons	Self-employed: business
	Self-employed Persons [Agriculture (Agricultural Labourers)], Self-employed Persons [Agriculture (Farmers (Who Cultivate Their Own Land*), Farmers/Cultivators (Who Cultivate On Lease Land#), Persons engaged in Farming Sector (Agricultural Labourers)	Self-employed: farm/agriculture
	Student	Student
	Unemployed	Unemployed
	Daily Wage Earner	Daily Wage Earner
	Retired	Home duties
	Other	Other
Reason for suicide	Bankruptcy or Indebtedness, Poverty, Unemployment	Economic
	Illness (AIDS/STD), Illness (Cancer), Illness (Paralysis), Illness (Insanity/Mental illness), Illness (Other Prolonged Illness), Drug Abuse/Alcoholic Addiction	Health
	Non-Settlement of Marriage, Dowry Related Issues, Extra Marital affairs, Divorce, Others	Marriage
	Impotency/Infertility, Death of Dear Person, Fall in Social Reputation, Ideological Causes/Hero Worshipping, Property dispute, Suspected/ Illicit Relation (Other than Extra Marital Affairs), Illegitimate Pregnancy (Other than Extra Marital Affairs), Professional/Career Problem	Personal/social
	Family problems	Family problems
	Love affair	Love affair
	Failure in Examination	Exam
	Physical Abuse (Rape, etc.)	Rape
	Causes not known	Unknown
	Other causes	Other
Means of suicide	Drowning	Drowning
	Hanging	Hanging
	Fire/self-immolation	Self-immolation
	Consuming sleeping pills	Overdose pills

	NCRB category	Re-categorisation
	Consuming insecticides	Insecticides
	Consuming other poison	Poison other
	From building, from other sites, jumping off moving trains/vehicles, coming under running vehicles/trains	Jumping
	Firearms, Self-inflicting injury, touching electric wire, other means	Other

2: Re-categorisation of the National Sample Survey (NSSO) categories of education, marital status and occupation for analysis.

	NSSO category	Re-categorisation
Education	Not literate, literate without any schooling, literate without any formal schooling, literate through TLC/ AEC, others	No education
	Below primary, primary	Class 1-5
	Upper primary/middle	Class 6-8
	Secondary, Higher secondary	Class 9 or higher
	Diploma/ certificate course up to secondary, Diploma/ certificate course up to higher secondary, Diploma/ certificate course graduation and above	
	Graduate, Post graduate & above	Graduate and above
Marital status	Never married	Never married
	Currently married	Currently married
	Widowed, Divorced/Separated	Previously married
Occupation	Attended domestic duties only	Housewife
	Attended domestic duties and was also engaged in free collection of goods (vegetables, roots, firewood, cattle feed, etc.), sewing, tailoring, weaving, etc	
	Worked as regular salaried/ wage employee	Professional/Salaried employee
	Worked in household. enterprise (self-employed): own account worker	Self-employed: business
	Worked in household. enterprise (self-employed): employer	
	Worked as casual wage labour: in public works	Daily Wage Earner
	Worked as casual wage labour: in other types of work	
	Attended educational institution	Student
	Did not work but was seeking and/or available for work	Unemployed
	Worked as helper enterprise (unpaid family worker)	Other
	Rentiers, pensioners , remittance recipients/ not able to work due to disability	
	Others (including begging, prostitution, etc.)	

3: Suicide death rate per 100,000 women for India and grouping of states by development status, 2014 to 2020.* Error bars denote 95% confidence interval.

India

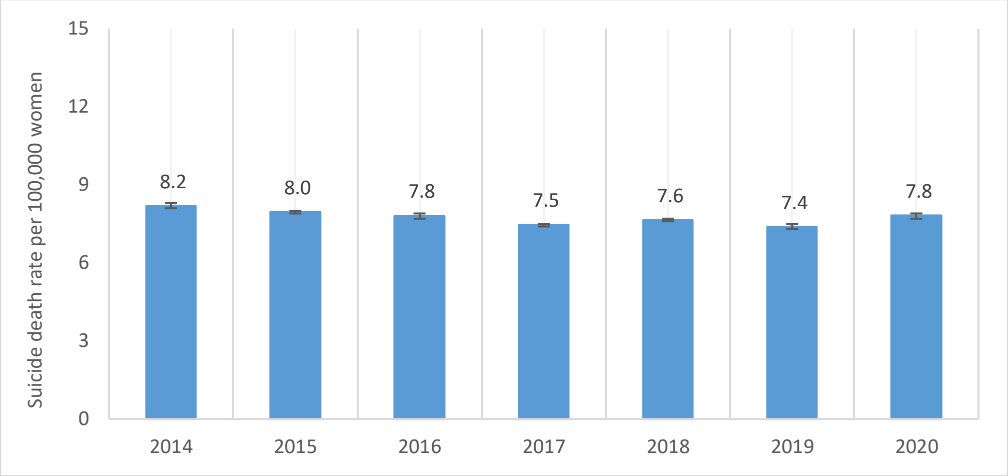

Less developed states

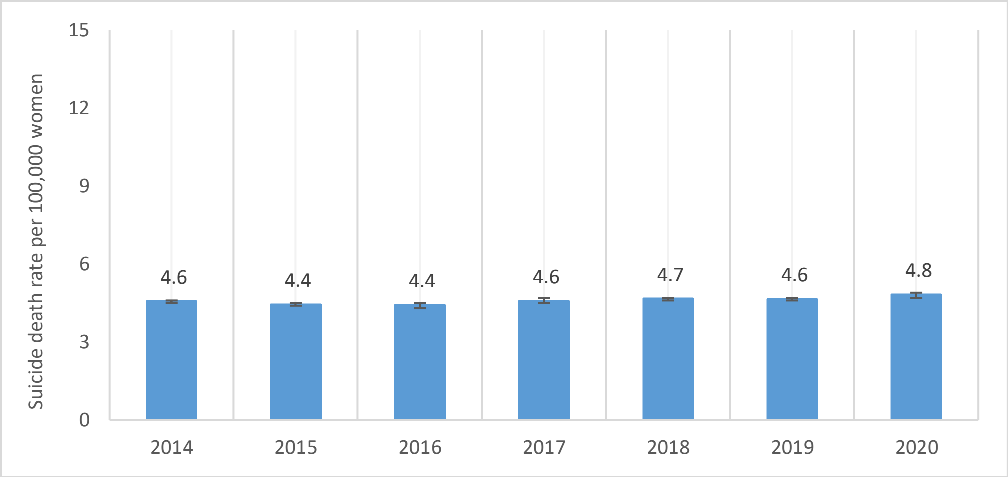

More developed states

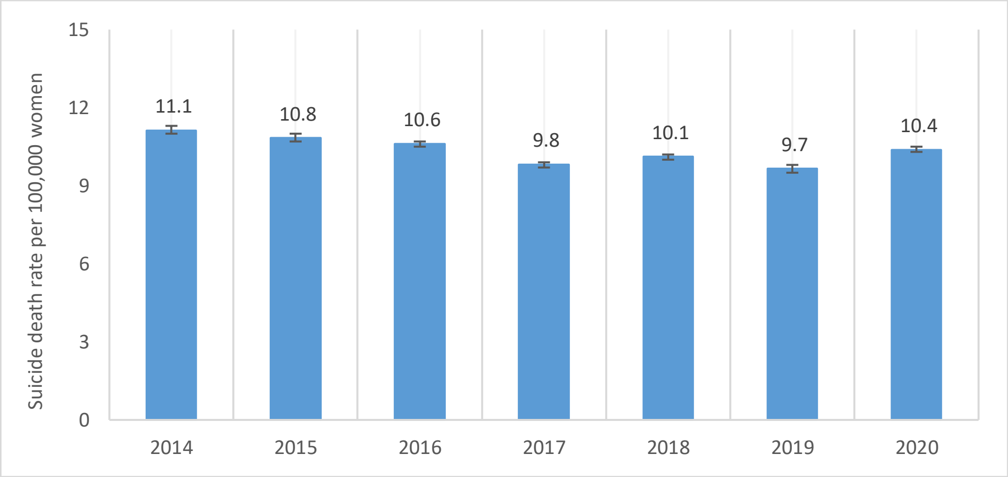

*Numerator from National Crimes Record Bureau and denominator from the Global Burden of Disease Study

4: Suicide death rate per 100,000 women in India and states, 2014 to 2020.*

	**Suicide death rate per 100,000 women population (95% Confidence Interval)**							**Percent change from 2014 to 2020 (95% Confidence Interval)**
	**2014**	**2015**	**2016**	**2017**	**2018**	**2019**	**2020**	
**India**	8·2 (8·1-8·3)	8·0 (7·9-8·0)	7·8 (7·7-7·9)	7·5 (7·4-7·5)	7·6 (7·6-7·7)	7·4 (7·3-7·5)	7·8 (7·7-7·9)	-4·5 (-5·8 to -3·2)
Less developed states	4·6 (4·5-4·6)	4·4 (4·4-4·5)	4·4 (4·3-4·5)	4·6 (4·5-4·7)	4·7 (4·6-4·7)	4·6 (4·6-4·7)	4·8 (4·7-4·9)	5·6 (3·2 to 8·1)
Arunachal Pradesh	0·2 (0·1-0·3)	0·2 (0·1-0·3)	0·2 (0·1-0·2)	0·1 (0·1-0·2)	0·2 (0·1-0·2)	0·1 (0·1-0·2)	0·2 (0·1-0·3)	-0·2 (-40·6 to 40·2)
Assam	7·8 (7·4-8·3)	6·4 (6·0-6·8)	7·2 (6·8-7·7)	4·9 (4·5-5·3)	4·9 (4·6-5·3)	4·8 (4·4-5·2)	6·2 (5·8-6·6)	-20·6 (-28·6 to -12·6)
Bihar	0·7 (0·6-0·8)	0·5 (0·5-0·6)	0·5 (0·4-0·5)	0·4 (0·4-0·5)	0·4 (0·4-0·5)	0·5 (0·5-0·6)	0·8 (0·7-0·9)	8·6 (-7·4 to 24·6)
Chhattisgarh	16·9 (16·2-17·7)	18·5 (17·7-19·3)	18·0 (17·2-18·7)	15·7 (15·0-16·4)	16·5 (15·8-17·3)	16·3 (15·6-17·0)	16·7 (16·0-17·4)	-1·2 (-7·3 to 4·9)
Jharkhand	3·0 (2·7-3·3)	1·9 (1·7-2·1)	2·9 (2·6-3·2)	2·2 (2·0-2·5)	2·8 (2·5-3·0)	3·9 (3·5-4·2)	5·0 (4·6-5·3)	66·7 (51.4 to 82.0)
Madhya Pradesh	12·1 (11·8-12·5)	12·9 (12·5-13·3)	13·2 (12·8-13·6)	13·4 (13·0-13·8)	12·9 (12·5-13·3)	12·5 (12·1-12·8)	13·6 (13·2-14·0)	11·9 (7·4 to 16·4)
Manipur	1·5 (0·8-2·1)	1·2 (0·6-1·8)	1·2 (0·6-1·7)	1·1 (0·6-1·7)	1·2 (0·6-1·8)	1·5 (0·9-2·1)	1·1 (0·6-1·7)	-21·4 (-79·9 to 37·1)
Meghalaya	2·0 (1·2-2·8)	3·3 (2·3-4·3)	3·3 (2·3-4·3)	2·7 (1·8-3·6)	2·8 (1·9-3·8)	2·4 (1·6-3·3)	4·4 (3·3-5·5)	124·5 (55·4 to 193·6)
Mizoram	3·4 (1·7-5·1)	4·6 (2·7-6·5)	4·9 (2·9-6·8)	3·0 (1·5-4·5)	2·4 (1·0-3·7)	3·1 (1·6-4·6)	3·8 (2·1-5·5)	12·3 (-57·2 to 81·7)
Nagaland	0·5 (0·0-1·0)	0·7 (0·1-1·2)	1·7 (0·8-2·6)	1·0 (0·3-1·8)	0·8 (0·2-1·4)	0·7 (0·1-1·2)	1·6 (0·7-2·5)	201·2 (4·6 to 397·8)
Odisha	9·9 (9·4-10·3)	9·1 (8·7-9·5)	8·0 (7·6-8·4)	12·0 (11·5-12·5)	11·8 (11·3-12·3)	10·2 (9·8-10·7)	8·4 (8·0-8·9)	-14·7 (-20·8 to -8·5)
Rajasthan	4·3 (4·0-4·5)	3·1 (2·9-3·3)	3·6 (3·4-3·9)	3·5 (3·3-3·7)	4·0 (3·8-4·2)	3·9 (3·7-4·1)	5·1 (4·8-5·3)	19·0 (10.9 to 27.0)
Sikkim	32·0 (25·1-38·9)	27·0 (20·7-33·3)	29·6 (23·1-36·2)	19·6 (14·3-24·9)	20·6 (15·2-26·0)	21·2 (15·7-26·7)	24·7 (18·8-30·6)	-22·8 (-51·0 to 5·5)
Tripura	16·8 (14·8-18·8)	16·6 (14·6-18·6)	13·5 (11·7-15·2)	14·2 (12·4-16·0)	13·4 (11·7-15·2)	13·6 (11·9-15·4)	14·6 (12·8-16·4)	-13·2 (-29·3 to 2·9)
Uttar Pradesh	1·8 (1·7-1·9)	1·9 (1·8-2·0)	1·7 (1·6-1·8)	2·1 (2·0-2·2)	2·2 (2·1-2·3)	2·4 (2·3-2·5)	1·7 (1·6-1·8)	-5·5 (-12·3 to 1·4)
Uttarakhand	1·3 (0·9-1·6)	2·9 (2·4-3·4)	1·2 (0·9-1·5)	2·6 (2·2-3·1)	3·6 (3·1-4·2)	3·7 (3·1-4·2)	5·4 (4·8-6·1)	326·9 (270·1 to 383·7)
More developed states	11·1 (11·0-11·3)	10·8 (10·7-11·0)	10·6 (10·5-10·7)	9·8 (9·7-9·9)	10·1 (10·0-10·2)	9·7 (9·5-9·8)	10·4 (10·3-10·5)	-6·7 (-8·3 to -5·2)
Andhra Pradesh	8·4 (8·0-8·7)	8·4 (8·1-8·8)	8·5 (8·2-8·9)	6·5 (6·2-6·8)	6·7 (6·4-7·0)	7·4 (7·0-7·7)	8·0 (7·6-8·4)	-4·2 (-10·4 to 2·1)
Delhi	9·5 (8·8-10·2)	9·0 (8·3-9·7)	9·2 (8·5-9·8)	10·2 (9·5-10·9)	9·8 (9·1-10·5)	9·9 (9·2-10·5)	11·3 (10·5-12·0)	18·9 (8.0 to 29.7)
Goa	13·4 (10·6-16·2)	12·4 (9·7-15·1)	10·5 (8·0-12·9)	12·2 (9·5-14·8)	9·1 (6·8-11·4)	8·7 (6·4-10·9)	11·1 (8·6-13·6)	-17·2 (-45·4 to 10·9)
Gujarat	10·1 (9·7-10·5)	9·6 (9·2-10·0)	10·0 (9·6-10·3)	9·4 (9·1-9·8)	9·4 (9·0-9·7)	9·0 (8·6-9·3)	9·2 (8·8-9·5)	-9·0 (-14·3 to -3·8)
Haryana	6·5 (6·0-6·9)	6·9 (6·4-7·4)	7·0 (6·5-7·4)	6·5 (6·1-7·0)	6·8 (6·3-7·2)	7·8 (7·2-8·3)	7·8 (7·3-8·3)	20·9 (10·1 to 31·7)
Himachal Pradesh	8·1 (7·1-9·1)	6·3 (5·4-7·2)	7·2 (6·3-8·2)	7·5 (6·6-8·5)	7·7 (6·7-8·6)	6·4 (5·5-7·2)	9·0 (8·0-10·0)	10·9 (-6·8 to 28·6)
Undivided Jammu & Kashmir	2·1 (1·7-2·5)	3·3 (2·8-3·8)	2·5 (2·1-3·0)	2·6 (2·1-3·0)	3·2 (2·7-3·7)	2·3 (1·9-2·7)	2·3 (1·9-2·7)	9·8 (-16·8 to 36·4)
Karnataka	12·0 (11·6-12·4)	12·1 (11·7-12·5)	10·9 (10·6-11·3)	11·2 (10·8-11·6)	11·1 (10·7-11·4)	10·0 (9·7-10·4)	10·6 (10·3-11·0)	-11·2 (-15·9 to -6·6)
Kerala	12·9 (12·3-13·4)	11·7 (11·2-12·2)	11·1 (10·5-11·6)	11·0 (10·5-11·5)	11·6 (11·1-12·2)	11·8 (11·2-12·3)	12·0 (11·5-12·6)	-6·5 -12.5 to -0.4)
Maharashtra	9·2 (8·9-9·4)	8·7 (8·4-9·0)	8·6 (8·3-8·8)	8·7 (8·4-8·9)	8·7 (8·5-9·0)	8·6 (8·4-8·9)	8·6 (8·4-8·9)	-6·0 (-10.0 to -2.0)
Punjab	2·0 (1·7-2·2)	1·9 (1·7-2·2)	2·4 (2·1-2·7)	2·6 (2·3-2·9)	2·8 (2·6-3·1)	4·2 (3·9-4·6)	4·9 (4·5-5·2)	148·8 (125.4 to 172.2)
Tamil Nadu	15·5 (15·1-15·9)	15·0 (14·6-15·4)	14·5 (14·1-14·9)	13·2 (12·9-13·6)	13·6 (13·2-14·0)	12·3 (11·9-12·6)	15·2 (14·8-15·6)	-2·1 (-5.9 to 1.7)
Telangana	18·4 (17·8-19·1)	17·4 (16·8-18·0)	16·4 (15·8-17·0)	14·1 (13·5-14·7)	13·4 (12·9-14·0)	12·4 (11·9-13·0)	13·1 (12·5-13·6)	-29·1 (-33.7 to -24.4)
West Bengal	13·5 (13·2-13·9)	13·6 (13·3-14·0)	13·7 (13·4-14·1)	11·4 (11·1-11·7)	13·0 (12·6-13·3)	11·7 (11·4-12·1)	12·3 (12·0-12·6)	-9·3 (-12.9 to -5.6)

* Numerator from National Crimes Record Bureau (NCRB) and denominator from the Global Burden of Disease Study

5. Distribution of deaths by suicide of women by their education level for Indian states as reported by the National Crimes Report Bureau, 2014 and 2020.

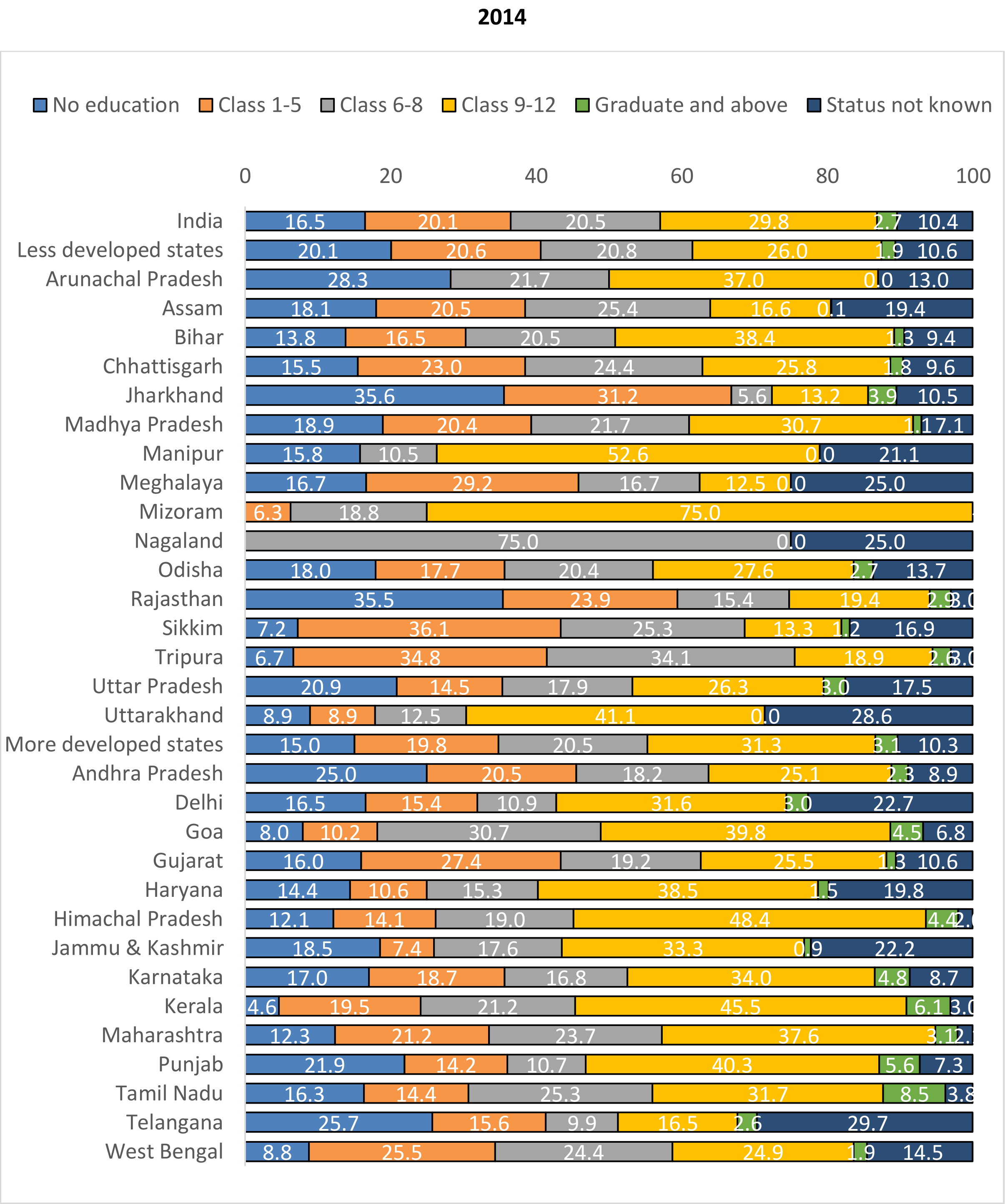

2020

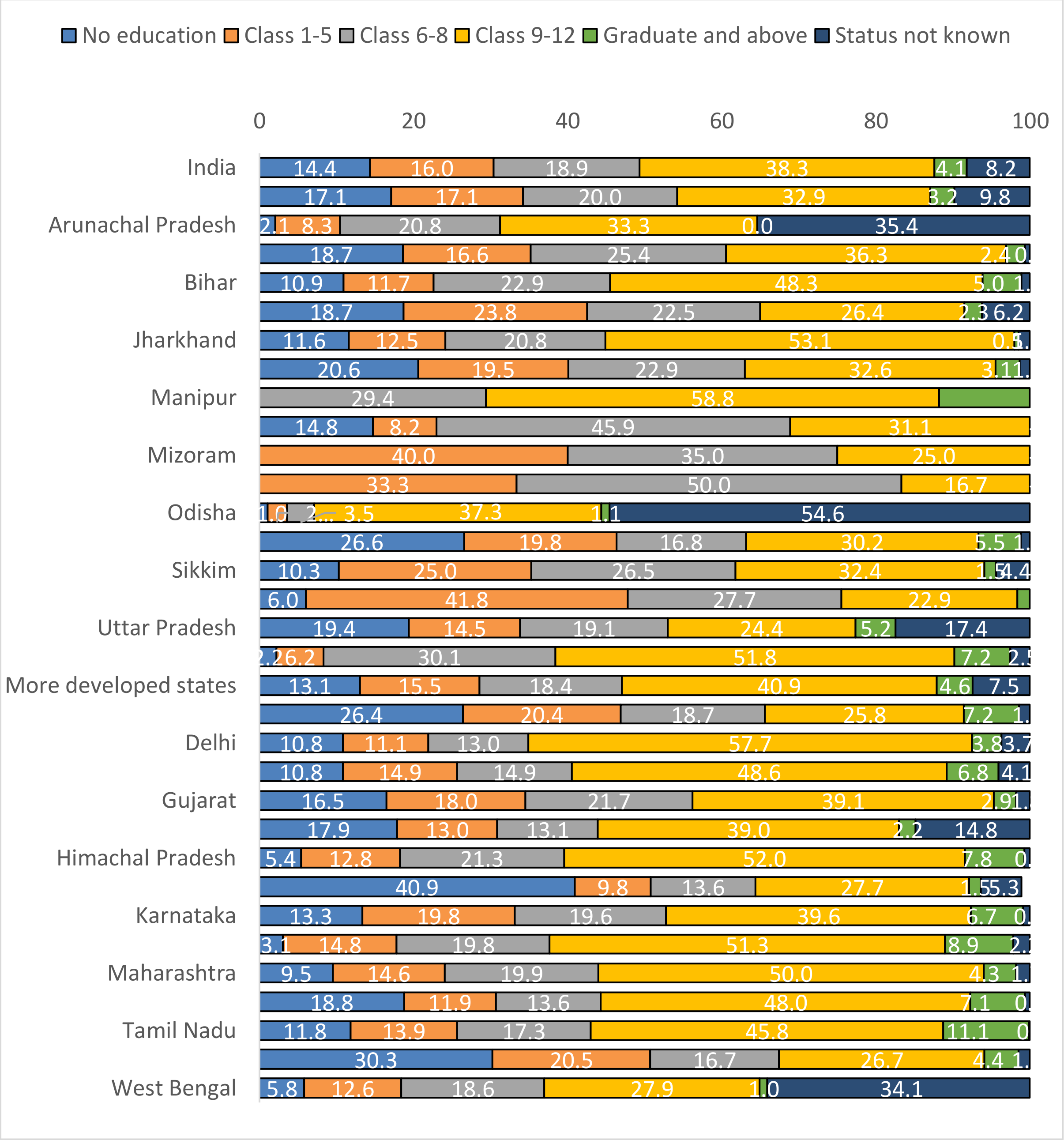
No education
Class 1-5
Class 6-8
Class 9-12
Graduate and above
Status not known
0
20
40
60
80
100
India
Arunachal Pradesh
Bihar
Jharkhand
Manipur
Mizoram
Odisha
Sikkim
Uttar Pradesh
More developed states
Delhi
Gujarat
Himachal Pradesh
Karnataka
Maharashtra
Tamil Nadu
West Bengal

6. Suicide death rate per 100,000 women for India and its states - overall, by level of education and marital status, 2020.

	Suicide death rate per 100,000 women (95% Confidence Interval)								
	**Overall***	**Education****					**Marital Status****		
		**No education**	**Class 1-5**	**Class 6-8**	**Class 9-12**	**Graduate or above**	**Never married**	**Currently married**	**Previously married**
**India**	**7·8 (7·7-7·9)**	**3.8 (3.7-3.9)**	**5.4 (5.2-5.5)**	**9.5 (9.3-9.7)**	**12.9 (12.7-13.1)**	**4.0 (3.9-4.2)**	**8·4 (8·2-8·5)**	**7·6 (7·5-7·7)**	**2·8 (2·7-2·9)**
Less developed states	**4·8 (4·7-4·9)**	**2.5 (2.4-2.6)**	**3.7 (3.6-3.9)**	**6.5 (6.3-6.8)**	**9.0 (8.7-9.2)**	**2.8 (2.6-3.1)**	**5·8 (5·6-6·0)**	**4·5 (4·4-4·6)**	**1·6 (1·5-1·8)**
Arunachal Pradesh	0·2 (0·1-0·3)	0.6 (-0.6-1.8)	2.3 (0.0-4.6)	7.4 (2.8-11.9)	9.1 (4.6-13.6)	0	10·0 (5·8-14·2)	3·6 (1·8-5·4)	0
Assam	6·2 (5·8-6·6)	6.2 (5.3-7.2)	3.5 (2.9-4.1)	7.8 (6.8-8.8)	8.3 (7.4-9.2)	3.4 (2.0-4.8)	8·3 (7·4-9·2)	6·0 (5·5-6·5)	1·7 (1·0-2·4)
Bihar	0·8 (0·7-0·9)	0.2 (0.2-0.3)	0.4 (0.3-0.5)	1.2 (0.9-1.5)	1.9 (1.6-2.2)	1.3 (0.7-1.9)	1·4 (1·2-1·6)	0·6 (0·5-0·6)	1·1 (0·6-1·5)
Chhattisgarh	16·7 (16·0-17·4)	10.5 (9.5-11.5)	14.6 (13.4-15.8)	20.3 (18.5-22.1)	23.0 (21.2-24.9)	7.4 (5.4-9.4)	21·9 (20·4-23·5)	15·7 (14·9-16·5)	4·9 (3·7-6·0)
Jharkhand	5·0 (4·6-5·3)	1.6 (1.2-1.9)	2.2 (1.7-2.6)	7.0 (5.9-8.1)	15.6 (14.1-17.2)	1.1 (0.0-2.2)	9·4 (8·4-10·4)	3·8 (3·4-4·2)	1·4 (0·8-1·9)
Madhya Pradesh	13·6 (13·2-14·0)	8.3 (7.8-8.8)	10.6 (10.0-11.3)	15.8 (14.8-16.7)	26.7 (25.4-28.0)	8.4 (7.0-9.7)	16·0 (15·2-16·8)	13·6 (13·1-14·1)	4·2 (3·5-5·0)
Manipur	1·1 (0·6-1·7)	0	0	1.5 (0.2-2.9)	1.8 (0.7-2.9)	1.3 (-0.5-3.0)	1·4 (0·4-2·4)	1·2 (0·4-1·9)	0
Meghalaya	4·4 (3·3-5·5)	9.1 (3.2-15.1)	1.0 (0.1-1.8)	8.4 (5.3-11.5)	5.9 (3.2-8.5)	0	6·5 (4·4-8·6)	3·0 (1·7-4·3)	1·7 (-0·6-4·0)
Mizoram	3·8 (2·1-5·5)	0	6.0 (1.8-10.1)	4.1 (1.1-7.2)	2.8 (0.3-5.2)	0	5·2 (2·0-8·4)	3·6 (1·4-5·8)	0
Nagaland	1·6 (0·7-2·5)	0	2.0 (0.0-4.0)	3.9 (0.8-7.0)	0.7 (-0.3-1.8)	0	2·1 (0·4-3·8)	1·1 (0·1-2·0)	4·9 (-4·7-14·4)
Odisha	8·4 (8·0-8·9)	0.3 (0.1-0.4)	0.7 (0.5-1.0)	1.9 (1.4-2.4)	17.1 (15.7-18.4)	1.8 (0.9-2.6)	2·9 (2·4-3·4)	1·2 (1·0-1·4)	0·2 (0·0-0·5)
Rajasthan	5·1 (4·8-5·3)	2.9 (2.7-3.2)	4.6 (4.1-5.1)	7.0 (6.2-7.8)	11.0 (10.1-12.0)	4.6 (3.7-5.6)	5·9 (5·4-6·5)	4·9 (4·6-5·2)	2·2 (1·7-2·8)
Sikkim	24·7 (18·8-30·6)	21.8 (5.6-37.9)	24.8 (13.0-36.6)	35.1 (18.9-51.2)	23.3 (13.6-33.1)	3.5 (-3.3-10.3)	26·4 (16·1-36·8)	24·4 (16·9-31·8)	8·0 (-7·7-23·7)
Tripura	14·6 (12·8-16·4)	6.7 (3.3-10.1)	15.2 (12.3-18.2)	13.7 (10.5-17.0)	25.9 (19.2-32.6)	5.0 (0.1-9.8)	16·1 (12·2-19·9)	16·0 (13·7-18·4)	0·6 (-0·6-1·8)
Uttar Pradesh	1·7 (1·6-1·8)	0.9 (0.8-1.0)	1.2 (1.1-1.4)	2.2 (1.9-2.4)	2.1 (1.9-2.3)	1.1 (0.9-1.4)	1·6 (1·4-1·7)	1·7 (1·6-1·8)	0·5 (0·4-0·7)
Uttarakhand	5·4 (4·8-6·1)	0.6 (0.1-1.1)	1.5 (0.8-2.3)	9.2 (7.2-11.2)	9.5 (8.0-11.1)	3.5 (1.9-5.0)	7·4 (5·9-8·8)	5·3 (4·5-6·1)	0
More developed states	**10·4 (10·3-10·5)**	**5.4 (5.3-5.6)**	**7.0 (6.8-7.2)**	**12.5 (12.2-12.9)**	**15.8 (15.5-16.0)**	**4.9 (4.6-5.1)**	**11·4 (11·2-11·7)**	**10·6 (10·4-10·7)**	**3·6 (3·4-3·8)**
Andhra Pradesh	8·0 (7·6-8·4)	4.9 (4.5-5.4)	8.7 (7.9-9.6)	12.8 (11.5-14.2)	9.8 (9.0-10.7)	10.2 (8.5-11.9)	9·3 (8·4-10·2)	9·4 (8·9-9·9)	0·9 (0·6-1·1)
Delhi	11·3 (10·5-12·0)	7.4 (5.9-8.8)	8.5 (6.8-10.1)	11.1 (9.1-13.1)	19.3 (17.6-21.0)	2.0 (1.3-2.6)	14·3 (12·7-15·9)	10·3 (9·4-11·1)	5·7 (3·6-7·9)
Goa	11·1 (8·6-13·6)	13.0 (4.0-22.0)	8.9 (3.6-14.1)	7.8 (3.2-12.3)	15.3 (10.3-20.3)	4.8 (0.6-9.1)	11·4 (6·0-16·9)	11·1 (8·0-14·2)	9·2 (2·4-15·9)
Gujarat	9·2 (8·8-9·5)	5.8 (5.3-6.4)	6.0 (5.5-6.6)	12.9 (11.8-13.9)	16.0 (15.0-17.0)	3.0 (2.3-3.7)	11·2 (10·4-12·0)	9·3 (8·8-9·7)	3·0 (2·4-3·7)
Haryana	7·8 (7·3-8·3)	4.7 (4.0-5.4)	5.4 (4.4-6.3)	6.9 (5.6-8.1)	12.2 (10.9-13.4)	1.5 (0.8-2.1)	9·5 (8·4-10·7)	7·4 (6·8-8·0)	1·6 (0·8-2·4)
Himachal Pradesh	9·0 (8·0-10·0)	2.4 (1.2-3.5)	5.6 (3.8-7.4)	13.0 (9.8-16.3)	14.3 (12.0-16.5)	6.0 (3.6-8.5)	10·5 (8·2-12·9)	9·9 (8·6-11·2)	1·2 (0·2-2·3)
Undivided Jammu & Kashmir	2·3 (1·9-2·7)	3.0 (2.2-3.8)	1.3 (0.6-1.9)	1.6 (0.8-2.3)	2.8 (1.9-3.7)	0.5 (-0.2-1.3)	3·5 (2·5-4·4)	1·6 (1·2-2·0)	0·3 (-0·3-0·9)
Karnataka	10·6 (10·3-11·0)	5.2 (4.7-5.7)	9.6 (8.8-10.4)	18.2 (16.7-19.6)	14.5 (13.7-15.4)	6.5 (5.5-7.4)	11·8 (10·9-12·6)	11·8 (11·3-12·3)	3·1 (2·6-3·7)
Kerala	12·0 (11·5-12·6)	6.9 (5.2-8.7)	8.0 (7.1-9.0)	12.6 (11.3-13.9)	17.7 (16.7-18.8)	3.9 (3.2-4.7)	11·8 (10·7-12·9)	13·5 (12·8-14·3)	5·5 (4·6-6·5)
Maharashtra	8·6 (8·4-8·9)	3.9 (3.5-4.3)	5.5 (5.1-5.9)	9.9 (9.2-10.5)	15.4 (14.8-16.1)	2.6 (2.2-3.1)	11·2 (10·6-11·8)	8·6 (8·3-8·9)	2·9 (2·5-3·4)
Punjab	4·9 (4·5-5·2)	5.1 (4.1-6.0)	2.7 (2.1-3.3)	4.7 (3.7-5.8)	7.1 (6.3-7.9)	2.2 (1.5-2.9)	6·8 (5·9-7·7)	4·7 (4·3-5·2)	0·8 (0·3-1·4)
Tamil Nadu	15·2 (14·8-15·6)	8.0 (7.4-8.6)	9.3 (8.6-10.0)	19.4 (18.1-20.6)	24.2 (23.2-25.1)	12.3 (11.2-13.4)	16·2 (15·3-17·0)	16·9 (16·3-17·4)	6·8 (6·1-7·5)
Telangana	13·1 (12·5-13·6)	11.0 (10.2-11.9)	14.9 (13.5-16.3)	22.8 (20.5-25.2)	12.4 (11.4-13.4)	6.3 (5.0-7.7)	11·4 (10·3-12·5)	14·7 (13·9-15·4)	6·0 (4·9-7·1)
West Bengal	12·3 (12·0-12·6)	2.8 (2.5-3.2)	5.2 (4.8-5.6)	11.7 (11.0-12.4)	17.8 (16.9-18.8)	1.7 (1.2-2.2)	11·3 (10·6-12·0)	10·4 (10·0-10·7)	3·1 (2·6-3·5)

*Numerator from National Crimes Record Bureau (NCRB) and denominator from the Global Burden of Disease Study

**Numerator from NCRB and denominator from the National Sample Survey

7. Suicide death rate per 100,000 women for India and its states-overall, by level of education and marital status in the year 2014.

	**Education***					**Marital status***		
	**No Education**	**Class 1-5**	**Class 6-8**	**Class 9-12**	**Graduate or above**	**Never married**	**Currently married**	**Previously married**
**India**	**3.9 (3.8-4.0)**	**6.7 (6.5-6.8)**	**11.1 (10.9-11.4)**	**13.3 (13.1-13.6)**	**3.1 (2.9-3.3)**	**7·5 (7·4-7·7)**	**8·1 (8·0-8·2)**	**3·6 (3·4-3·7)**
Less developed states	***2.4 (2.3-2.5)***	***4.2 (4.0-4.4)***	***6.9 (6.7-7.2)***	***9.0 (8.7-9.3)***	***1.9 (1.6-2.1)***	***4·7 (4·6-4·9)***	***4·7 (4·6-4·8)***	***2·2 (2·0-2·4)***
Arunachal Pradesh	7.8 (3.6-12.1)	0	8.3 (3.2-13.5)	13.5 (7.1-19.9)	0	9·3 (5·3-13·2)	5·4 (2·8-7·9)	0
Assam	6.9 (5.9-7.8)	4.5 (3.9-5.2)	9.3 (8.2-10.4)	6.8 (5.8-7.9)	0.2 (-0.2-0.6)	11·2 (10·0-12·4)	6·1 (5·6-6·7)	0·3 (0·0-0·6)
Bihar	0.2 (0.1-0.3)	0.5 (0.4-0.6)	1.2 (0.9-1.5)	1.9 (1.6-2.3)	0.4 (0.0-0.9)	1·0 (0·8-1·2)	0·6 (0·5-0·7)	0·2 (0·0-0·4)
Chhattisgarh	7.1 (6.3-7.9)	13.3 (12.1-14.5)	23.9 (21.7-26.0)	31.0 (28.3-33.8)	12.7 (8.4-17.0)	18·1 (16·5-19·7)	17·1 (16·2-18·0)	8·1 (6·3-9·8)
Jharkhand	2.4 (2.0-2.8)	4.2 (3.5-4.9)	1.2 (0.7-1.6)	2.7 (2.0-3.4)	2.6 (1.3-3.9)	2·7 (2·2-3·3)	2·3 (1·9-2·6)	1·8 (1·1-2·5)
Madhya Pradesh	5.5 (5.1-5.9)	10.1 (9.4-10.9)	16.1 (15.0-17.2)	32.6 (30.7-34.5)	2.3 (1.6-3.0)	11·6 (10·8-12·3)	12·9 (12·4-13·4)	3·2 (2·5-3·8)
Manipur	1.4 (-0.2-2.9)	0	0.7 (-0.3-1.6)	2.4 (0.9-3.9)	0	1·5 (0·3-2·7)	1·7 (0·8-2·6)	0
Meghalaya	5.1 (0.1-10.0)	1.2 (0.3-2.0)	1.8 (0.0-3.7)	1.2 (-0.2-2.6)	0	2·2 (0·9-3·4)	1·0 (0·2-1·8)	3·0 (-0·4-6·4)
Mizoram	0	0.6 (-0.6-1.9)	1.9 (-0.3-4.1)	11.1 (4.8-17.4)	0	2·8 (0·3-5·3)	2·4 (0·5-4·4)	8·7 (0·2-17·1)
Nagaland	0	0	1.3 (-0.2-2.9)	0	0	0·9 (-0·1-2·0)	0·2 (-0·2-0·7)	0
Odisha	5.0 (4.5-5.6)	6.7 (6.0-7.5)	10.2 (9.2-11.2)	18.2 (16.6-19.8)	6.8 (4.9-8.7)	9·4 (8·6-10·3)	6·5 (6·1-7·0)	7·5 (6·3-8·8)
Rajasthan	3.1 (2.8-3.4)	4.5 (4.0-5.0)	5.3 (4.5-6.0)	7.7 (6.7-8.7)	2.4 (1.6-3.2)	4·0 (3·5-4·5)	4·3 (4·0-4·6)	1·8 (1·3-2·3)
Sikkim	15.4 (3.1-27.7)	46.4 (29.8-63.0)	30.6 (17.5-43.7)	16.1 (6.6-25.6)	5.3 (-5.1-15.7)	19·4 (10·2-28·6)	36·4 (27·0-45·7)	16·1 (-6·2-38·3)
Tripura	7.1 (3.8-10.4)	12.9 (10.3-15.5)	22.4 (17.9-27.0)	30.2 (21.9-38.5)	14.3 (3.7-24.8)	21·9 (17·3-26·5)	13·9 (11·6-16·2)	16·9 (11·0-22·7)
Uttar Pradesh	0.9 (0.8-1.0)	1.2 (1.0-1.3)	2.4 (2.2-2.7)	3.1 (2.8-3.4)	0.8 (0.6-1.0)	1·4 (1·2-1·5)	2·0 (1·9-2·1)	0·3 (0·2-0·4)
Uttarakhand	0.3 (0.0-0.7)	0.5 (0.1-0.9)	0.9 (0.2-1.5)	2.9 (1.7-4.0)	0	2·2 (1·3-3·1)	0·7 (0·4-1·0)	0
More developed states	***5.8 (5.6-5.9)***	***8.8 (8.6-9.0)***	***15.1 (14.7-15.4)***	***16.1 (15.7-16.4)***	***3.7 (3.5-4.0)***	***10·5 (10·2-10·7)***	***11·3 (11·1-11·5)***	***4·5 (4·3-4·7)***
Andhra Pradesh	5.0 (4.5-5.4)	7.6 (6.9-8.4)	14.2 (12.7-15.7)	10.9 (9.9-11.8)	3.4 (2.4-4.4)	6·7 (6·0-7·5)	9·0 (8·5-9·5)	3·2 (2·6-3·8)
Delhi	12.5 (10.1-14.8)	7.0 (5.6-8.4)	7.7 (5.9-9.4)	9.9 (8.5-11.2)	1.2 (0.7-1.8)	12·1 (10·5-13·7)	8·2 (7·3-9·0)	3·6 (2·1-5·1)
Goa	9.5 (2.5-16.6)	5.5 (1.9-9.1)	24.3 (15.2-33.5)	18.2 (12.2-24.2)	3.4 (0.1-6.8)	13·6 (8·3-19·0)	14·1 (10·5-17·7)	6·5 (0·1-12·9)
Gujarat	5.2 (4.7-5.7)	8.8 (8.1-9.4)	14.9 (13.6-16.1)	15.3 (14.1-16.4)	1.8 (1.2-2.3)	9·4 (8·6-10·2)	10·4 (9·9-10·8)	5·8 (4·9-6·7)
Haryana	3.0 (2.4-3.6)	3.1 (2.4-3.9)	7.5 (6.1-9.0)	10.4 (9.1-11.6)	0.9 (0.4-1.5)	4·6 (3·8-5·4)	5·4 (4·8-5·9)	3·5 (2·3-4·7)
Himachal Pradesh	4.5 (2.9-6.1)	5.3 (3.6-7.1)	12.1 (8.6-15.6)	11.8 (9.7-13.9)	3.3 (1.3-5.2)	5·6 (3·9-7·2)	9·7 (8·4-11·1)	1·7 (0·3-3·0)
Undivided Jammu & Kashmir	1.0 (0.6-1.4)	0.8 (0.3-1.4)	2.4 (1.3-3.5)	3.8 (2.6-5.1)	0.3 (-0.3-0.8)	2·6 (1·8-3·4)	1·1 (0·7-1·5)	0·3 (-0·3-0·9)
Karnataka	6.0 (5.5-6.5)	10.8 (9.9-11.6)	14.3 (13.1-15.5)	18.3 (17.2-19.3)	5.9 (4.9-6.9)	12·7 (11·8-13·5)	11·9 (11·3-12·4)	2·8 (2·3-3·3)
Kerala	8.7 (6.9-10.4)	9.6 (8.6-10.5)	12.3 (11.2-13.5)	19.5 (18.2-20.7)	4.6 (3.7-5.5)	14·0 (12·7-15·4)	14·2 (13·4-14·9)	5·5 (4·6-6·4)
Maharashtra	4.9 (4.4-5.3)	7.5 (7.1-8.0)	12.1 (11.4-12.9)	15.7 (15.0-16.5)	1.8 (1.4-2.2)	9·6 (9·1-10·2)	10·0 (9·7-10·4)	3·4 (3·0-3·9)
Punjab	1.5 (1.1-1.9)	1.5 (1.0-2.0)	1.8 (1.1-2.5)	2.6 (2.1-3.2)	0.9 (0.4-1.4)	1·6 (1·2-2·0)	2·2 (1·8-2·5)	0·2 (0·0-0·5)
Tamil Nadu	9.1 (8.5-9.7)	9.1 (8.5-9.8)	25.2 (23.9-26.6)	24.3 (23.1-25.4)	8.8 (7.8-9.8)	14·0 (13·2-14·9)	16·7 (16·1-17·3)	4·9 (4·3-5·5)
Telangana	10.4 (9.6-11.1)	15.2 (13.8-16.6)	28.5 (25.2-31.7)	13.8 (12.6-15.0)	6.4 (4.8-8.0)	12·6 (11·5-13·8)	16·9 (16·1-17·7)	10·5 (9·2-11·8)
West Bengal	4.1 (3.8-4.5)	11.1 (10.5-11.7)	17.9 (17.0-18.9)	22.6 (21.4-23.8)	3.6 (2.9-4.3)	14·1 (13·3-14·9)	12·9 (12·4-13·3)	5·6 (4·9-6·2)

*Numerator from National Crimes Record Bureau and denominator from the National Sample Survey

8. Distribution of marital status of women who died by suicide from 2014 to 2020 for India and its states as reported in the National Crimes Record Bureau Report. NM denotes never married, CM currently married, PM previously married, and OTH other.

	2014				2015				2016			
	**NM**	**CM**	**PM**	**OTH**	**NM**	**CM**	**PM**	**OTH**	**NM**	**CM**	**PM**	**OTH**
**India**	**23·1**	**63·6**	**4·8**	**8·5**	**23·1**	**67·3**	**4·4**	**5·2**	**24·3**	**64·4**	**4·3**	**7·1**
Less developed states	**25·9**	**61·1**	**3·7**	**9·3**	**24·5**	**64·8**	**3·8**	**6·9**	**26·9**	**62·7**	**3·9**	**6·5**
Arunachal Pradesh	45·7	37·0	0	17·4	47·8	28·3	0	23·9	48·8	39·5	0	11·6
Assam	33·0	52·8	0·4	13·8	31·5	66·0	2·1	0·5	32·7	65·2	1·5	0·6
Bihar	36·7	54·5	1·3	7·4	43·4	45·1	1·3	10·2	42·3	45·7	12·0	0
Chhattisgarh	26·9	66·3	4·4	2·3	26·4	59·9	4·8	8·9	30·7	56·2	4·3	8·8
Jharkhand	22·2	49·3	6·1	22·4	28·4	63·1	7·1	1·5	18·6	62·5	3·6	15·3
Madhya Pradesh	24·4	68·6	2·6	4·4	23·6	73·0	2·7	0·7	25·6	67·4	4·0	2·9
Manipur	31·6	68·4	0	0	25·0	75·0	0	0	25·0	75·0	0	0
Meghalaya	45·8	25·0	12·5	16·7	41·5	51·2	4·9	2·4	45·2	45·2	9·5	0
Mizoram	31·3	37·5	25·0	6·3	27·3	40·9	22·7	9·1	33·3	37·5	25·0	4·2
Nagaland	75·0	25·0	0	0	80·0	20·0	0	0	38·5	38·5	23·1	0
Odisha	24·2	42·6	7·6	25·7	19·2	49·7	6·8	24·2	28·9	44·9	2·9	23·3
Rajasthan	23·3	66·2	4·2	6·4	21·8	72·0	2·0	4·2	21·0	73·6	1·6	3·9
Sikkim	20·5	69·9	2·4	7·2	40·8	47·9	9·9	1·4	32·9	60·8	6·3	0
Tripura	32·2	52·6	11·9	3·3	32·5	62·4	3·7	1·5	25·1	67·7	7·2	0
Uttar Pradesh	23·6	68·5	1·3	6·7	20·8	68·1	3·6	7·4	22·1	70·5	5·0	2·4
Uttarakhand	45·7	37·0	0	17·4	24·8	62·8	4·7	7·8	26·4	73·6	0	0
More developed states	**21·9**	**64·7**	**5·3**	**8·1**	**22·5**	**68·4**	**4·6**	**4·5**	**23·1**	**65·0**	**4·5**	**7·4**
Andhra Pradesh	18·6	67·3	5·7	8·4	18·7	61·1	6·0	14·2	18·8	60·8	3·6	16·8
Delhi	33·7	56·3	3·1	6·9	32·9	60·6	0·6	5·9	32·2	60·2	1·0	6·5
Goa	28·4	65·9	4·5	1·1	29·3	63·4	4·9	2·4	30·0	64·3	5·7	0
Gujarat	21·1	69·2	5·9	3·8	24·3	71·0	3·7	1·0	24·2	69·7	4·9	1·3
Haryana	19·1	53·7	4·7	22·5	25·5	58·6	3·8	12·1	20·4	66·4	1·6	11·6
Himachal Pradesh	17·7	75·4	2·4	4·4	24·5	58·7	10·7	6·1	18·1	63·9	13·2	4·8
Undivided Jammu & Kashmir	37·0	32·4	0·9	29·6	29·4	42·9	0	27·6	25·6	39·8	0	34·6
Karnataka	25·1	60·0	3·6	11·2	21·7	71·1	2·8	4·4	21·3	65·3	3·2	10·2
Kerala	21·6	69·9	7·1	1·4	21·0	67·8	10·0	1·2	20·1	70·6	7·2	2·1
Maharashtra	24·6	70·2	4·6	0·5	25·2	68·9	5·4	0·5	26·8	68·6	3·5	1·1
Punjab	21·5	70·4	1·3	6·9	21·9	76·4	1·3	0·4	25·6	71·0	0·7	2·7
Tamil Nadu	21·0	66·1	4·9	8·1	21·8	70·5	5·4	2·2	23·5	70·6	5·3	0·6
Telangana	15·4	57·6	8·3	18·7	17·5	69·1	6·4	7·0	28·7	58·8	6·8	5·7
West Bengal	22·4	63·0	5·0	9·6	22·9	68·7	2·3	6·1	19·3	58·5	4·3	17·9

	2017				2018				2019				2020			
	**NM**	**CM**	**PM**	**OTH**	**NM**	**CM**	**PM**	**OTH**	**NM**	**CM**	**PM**	**OTH**	**NM**	**CM**	**PM**	**OTH**
**India**	**24·3**	**65·3**	**4·3**	**6·1**	**24·8**	**65·4**	**4·2**	**5·6**	**27·0**	**62·5**	**4·3**	**6·2**	**26·6**	**63·1**	**3·6**	**6·7**
Less developed states	**26·7**	**60·0**	**3·1**	**10·2**	**27·1**	**62·6**	**2·3**	**8·1**	**30·9**	**54·6**	**2·9**	**11·5**	**30·7**	**56·2**	**2·4**	**10·8**
Arunachal Pradesh	40·0	56·7	0	3·3	50·0	36·1	0	13·9	48·4	45·2	0	6·5	45·8	31·3	0	22·9
Assam	28·0	58·3	4·6	9·0	26·5	64·7	4·1	4·7	33·3	56·4	9·8	0·4	34·3	62·7	2·6	0·3
Bihar	38·5	55·2	6·3	0	39·5	48·6	10·3	1·6	43·9	50·8	5·3	0	41·1	51·4	6·7	0·8
Chhattisgarh	32·3	54·7	3·1	9·9	33·4	60·1	3·2	3·2	34·8	50·8	2·8	11·6	34·5	59·3	3·1	3·1
Jharkhand	47·5	48·5	3·4	0·6	36·5	58·1	4·4	1·0	39·5	52·2	7·4	0·9	45·5	50·1	2·8	1·7
Madhya Pradesh	26·7	68·3	4·5	0·5	28·5	68·4	2·9	0·2	29·7	67·5	2·5	0·3	32·5	64·9	2·3	0·3
Manipur	43·8	56·3	0	0	41·2	58·8	0	0	59·1	40·9	0	0	41·2	58·8	0	0
Meghalaya	52·8	36·1	2·8	8·3	44·7	39·5	7·9	7·9	54·5	42·4	3·0	0	60·7	34·4	3·3	1·6
Mizoram	60·0	33·3	6·7	0	50·0	41·7	8·3	0	56·3	43·8	0	0	50·0	50·0	0	0
Nagaland	25·0	75·0	0	0	16·7	83·3	0	0	80·0	20·0	0	0	50·0	41·7	8·3	0
Odisha	19·2	38·4	0·8	41·6	20·1	47·1	0	32·8	31·1	10·9	0·2	57·9	8·9	9·3	0·3	81·5
Rajasthan	27·1	69·3	2·0	1·6	23·0	65·4	1·1	10·5	28·9	59·7	2·6	8·8	29·5	65·3	3·4	1·8
Sikkim	50·9	45·3	3·8	0	42·9	53·6	3·6	0	27·6	70·7	1·7	0	36·8	60·3	1·5	1·5
Tripura	32·4	61·3	6·3	0	38·3	61·2	0·4	0	25·4	74·1	0·4	0	27·3	72·3	0·4	0
Uttar Pradesh	21·4	71·8	2·2	4·6	21·6	71·6	1·7	5·1	25·6	67·0	3·4	4·0	29·2	61·8	2·2	6·8
Uttarakhand	41·3	55·4	0	3·3	34·3	59·9	0	5·8	33·7	65·2	1·1	0	35·5	62·0	0	2·5
More developed states	**23·2**	**67·7**	**4·9**	**4·2**	**23·8**	**67·1**	**5·2**	**4·0**	**25·1**	**66·9**	**5·0**	**3·0**	**24·5**	**66·9**	**4·2**	**4·4**
Andhra Pradesh	20·6	71·8	3·6	4·0	20·2	71·5	4·1	4·2	27·1	64·6	7·8	0·5	22·6	74·6	1·8	1·0
Delhi	29·1	55·1	3·0	12·8	35·3	52·2	1·6	10·8	36·6	52·1	6·5	4·8	34·6	60·6	3·1	1·7
Goa	32·9	58·5	8·5	0	27·9	65·6	3·3	3·3	29·3	70·7	0	0	23·0	66·2	9·5	1·4
Gujarat	24·0	71·0	4·2	0·7	25·2	69·0	5·3	0·5	25·3	70·0	4·6	0·1	27·6	69·1	3·0	0·3
Haryana	28·8	65·5	3·3	2·3	24·7	68·8	3·1	3·4	22·3	70·4	1·2	6·2	28·5	64·8	1·6	5·1
Himachal Pradesh	23·3	66·7	10·0	0	33·6	65·2	1·2	0	24·6	69·6	5·8	0	26·7	71·6	1·7	0
Undivided Jammu & Kashmir	31·2	56·5	4·3	8·0	51·4	34·9	0	13·7	48·8	43·3	1·6	6·3	40·9	46·2	0·8	12·1
Karnataka	22·6	70·2	4·9	2·3	22·0	72·3	3·2	2·4	23·3	70·6	4·3	1·8	24·7	69·8	4·3	1·2
Kerala	20·7	72·5	5·5	1·4	19·0	72·9	6·9	1·2	19·7	72·1	6·7	1·5	21·3	70·4	7·2	1·1
Maharashtra	26·8	69·7	3·2	0·4	25·6	70·5	3·0	0·8	26·8	69·1	3·5	0·6	29·0	66·6	3·8	0·6
Punjab	23·5	70·6	3·7	2·1	26·8	68·2	4·5	0·6	26·0	70·5	2·0	1·5	31·9	66·3	1·6	0·2
Tamil Nadu	22·2	73·1	4·3	0·3	24·9	69·9	4·5	0·7	28·1	65·9	6·0	0·1	24·0	69·1	6·8	0·2
Telangana	16·9	72·0	9·4	1·7	21·1	66·4	11·2	1·3	21·0	64·0	12·5	2·5	20·1	73·6	5·2	1·1
West Bengal	23·7	54·6	5·8	15·9	21·9	57·9	6·9	13·3	22·8	63·7	2·4	11·1	19·5	56·7	2·8	20·9

9. Suicide death rate per 100,000 women for never married and currently married women for the states of India, 2014.*

Never married women

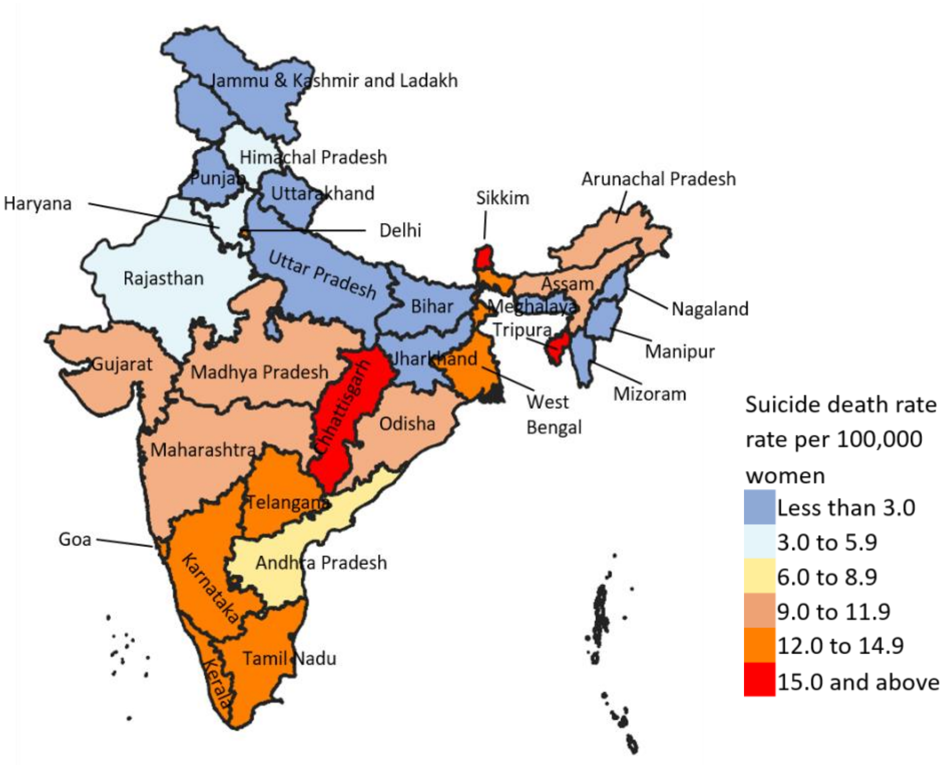

Currently married women

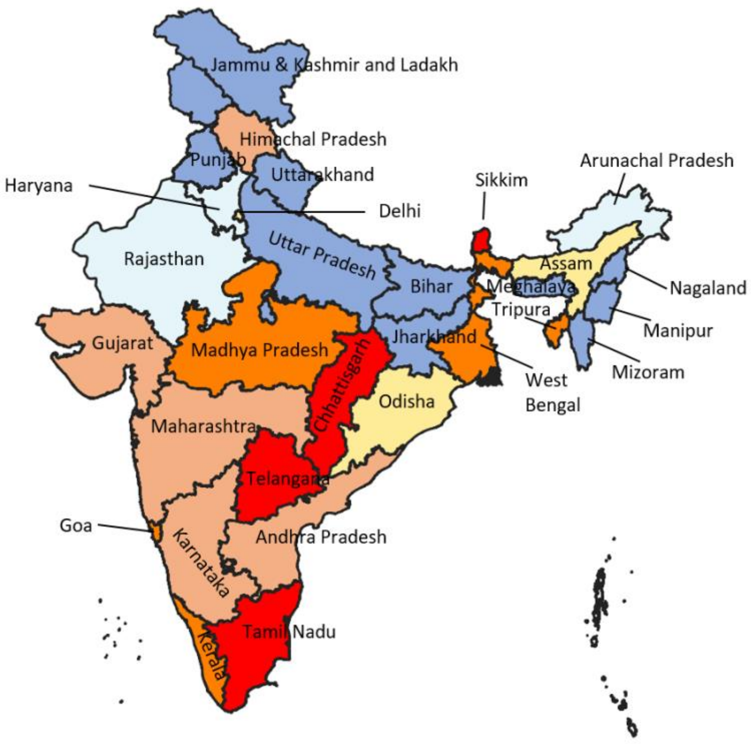

*Numerator from National Crimes Record Bureau and denominator from the National Sample Survey

10. Suicide death rate per 100,000 women for India and state categories by occupation, 2020.*

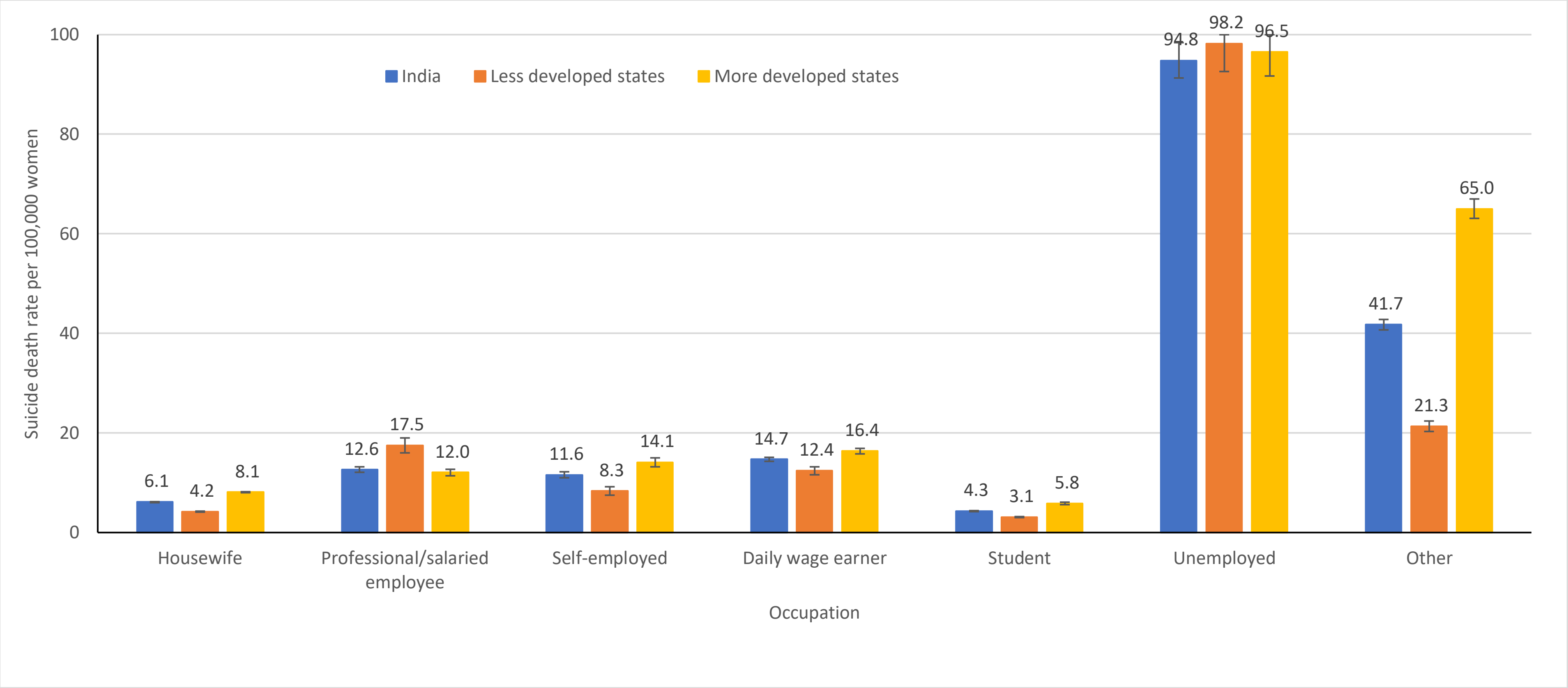

*Numerator from National Crimes Record Bureau and denominator from the National Sample Survey

11. Age-specific suicide death rate per 100,000 women for India, 2014 to 2020.*

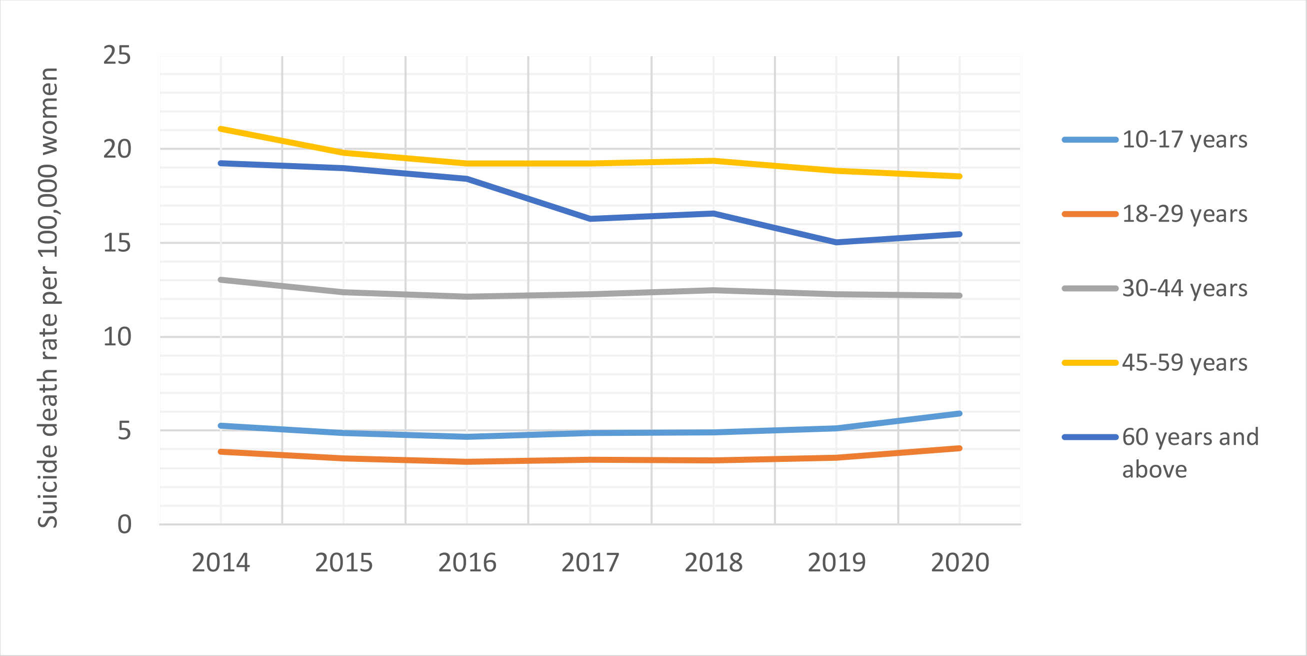

*Numerator from National Crimes Record Bureau and denominator from the Global Burden of Disease Study

12. Distribution of means of suicide death among women from 2014 to 2020 for India and its states as reported in the National Crimes Record Bureau Report. HG denotes hanging, IC insecticide consumption, SI self-immolation, CP consumption of poison, OTH other, DR drowning, JP jumping, and OP overdose of pills.

	2014								2015								2016							
	**HG**	**IC**	**SI**	**CP**	**OTH**	**DR**	**JP**	**OP**	**HG**	**IC**	**SI**	**CP**	**OTH**	**DR**	**JP**	**OP**	**HG**	**IC**	**SI**	**CP**	**OTH**	**DR**	**JP**	**OP**
**India**	36.8	10.7	13.1	15.5	14.9	6.3	2.2	0.6	40.7	16.8	13.9	11.1	8.8	5.7	2.5	0.6	41.7	15.7	13.0	10.6	11.3	4.9	2.2	0.6
Less developed states	39.5	8.9	12.5	14.3	16.5	5.6	2.0	0.8	41.0	11.8	14.6	12.8	11.6	5.2	2.6	0.5	46.9	13.3	12.3	10.1	10.6	4.0	2.1	0.8
Arunachal Pradesh	78.3	4.3	0	6.5	10.9	0	0	0	65.2	2.2	2.2	2.2	21.7	6.5	0	0	74.4	4.7	7.0	0	14.0	0	0	0
Assam	52.7	1.2	4.7	15.0	14.2	10.2	2.0	0	61.2	7.4	6.5	0	1.3	15.6	7.6	0.5	62.0	9.4	2.8	1.8	9.4	10.3	3.3	0.9
Bihar	34.3	11.4	13.1	6.1	22.6	9.1	1.0	2.4	40.7	9.7	9.3	8.4	19.0	7.1	0.4	5.3	51.0	28.4	2.9	6.7	3.4	2.4	3.4	1.9
Chhattisgarh	38.0	15.0	21.9	13.9	6.3	1.8	2.8	0.3	33.2	12.9	24.5	9.3	13.1	3.2	3.3	0.4	37.9	12.5	21.4	9.2	14.3	2.1	2.5	0.1
Jharkhand	52.0	2.7	1.2	2.7	32.7	5.9	2.4	0.5	45.5	17.5	17.5	0	8.6	5.2	3.7	1.9	51.3	2.4	8.1	11.7	16.0	5.0	2.9	2.6
Madhya Pradesh	38.5	13.4	17.0	13.6	7.8	7.8	0.8	1.1	39.6	19.2	18.3	8.1	8.0	5.8	0.8	0.3	43.1	17.4	17.7	11.9	4.8	3.7	1.1	0.4
Manipur	84.2	0	0	10.5	5.3	0	0	0	56.3	37.5	0	0	6.3	0	0	0	56.3	37.5	0	0	6.3	0	0	0
Meghalaya	75.0	0	8.3	4.2	12.5	0	0	0	80.5	0	12.2	4.9	0	0	2.4	0	78.6	0	11.9	2.4	2.4	0	0	4.8
Mizoram	100.0	0	0	0	0	0	0	0	90.9	4.5	0	0	0	0	4.5	0	75.0	0	4.2	0	16.7	0	4.2	0
Nagaland	100.0	0	0	0	0	0	0	0	100.0	0	0	0	0	0	0	0	92.3	0	0	0	0	7.7	0	0
Odisha	30.9	7.4	3.1	29.0	23.1	3.4	1.5	1.5	31.4	6.6	5.4	42.3	11.8	1.2	1.3	0	37.5	16.2	1.4	20.1	21.3	1.6	1.4	0.4
Rajasthan	31.4	5.7	8.3	8.8	34.5	6.5	4.2	0.7	39.0	6.2	7.4	13.0	19.2	8.7	5.9	0.5	44.6	9.2	6.7	8.2	20.4	6.7	4.1	0.2
Sikkim	79.5	1.2	2.4	1.2	13.3	2.4	0	0	81.7	0	1.4	0	15.5	0	1.4	0	86.1	0	1.3	0	10.1	2.5	0	0
Tripura	48.5	2.2	15.9	14.1	14.4	0.4	4.4	0	62.0	9.2	14.8	9.6	1.8	1.8	0.7	0	60.1	22.0	13.5	1.8	0	1.3	1.3	0
Uttar Pradesh	42.2	3.0	13.5	8.6	25.7	4.4	2.5	0.1	46.7	3.8	13.1	8.3	20.6	3.0	3.3	1.2	63.7	5.4	9.4	5.6	6.1	4.1	2.6	3.2
Uttarakhand	41.1	5.4	0	28.6	16.1	1.8	7.1	0	36.4	12.4	1.6	21.7	14.7	12.4	0.8	0	34.0	39.6	1.9	15.1	1.9	5.7	1.9	0
More developed states	35.3	11.5	13.4	16.1	14.2	6.6	2.2	0.6	40.4	18.9	13.6	10.4	7.6	6.0	2.5	0.6	39.3	16.8	13.3	10.9	11.6	5.4	2.2	0.5
Andhra Pradesh	24.5	11.2	14.4	27.2	11.5	5.9	3.5	1.9	28.2	31.8	17.2	6.4	5.7	4.6	4.9	1.0	33.0	19.9	19.7	11.0	8.7	4.4	2.0	1.3
Delhi	66.9	1.6	1.8	7.5	17.0	0.1	3.9	1.2	68.7	2.2	1.5	8.6	17.6	0.2	1.2	0	69.0	0.4	0.9	8.6	16.5	0	4.6	0
Goa	58.0	3.4	6.8	4.5	1.1	23.9	2.3	0	62.2	0	8.5	3.7	8.5	15.9	1.2	0	60.0	4.3	5.7	10.0	2.9	17.1	0	0
Gujarat	31.5	14.0	17.5	17.7	9.9	6.3	3.0	0.1	33.6	27.8	18.5	6.6	3.8	6.2	3.0	0.6	35.3	23.6	19.5	8.2	2.9	6.9	3.2	0.4
Haryana	32.5	4.1	6.8	25.7	23.8	3.5	2.8	0.9	21.7	5.9	7.2	24.3	16.7	12.7	11.3	0.1	34.1	8.3	9.1	17.9	16.6	5.4	7.9	0.7
Himachal Pradesh	22.6	19.8	6.0	27.0	18.5	5.2	0.8	0	20.4	35.7	1.5	23.0	14.8	2.6	2.0	0	27.3	22.5	4.0	32.2	4.8	6.2	3.1	0
Undivided Jammu & Kashmir	8.3	21.3	5.6	35.2	23.1	2.8	3.7	0	14.1	12.9	6.5	29.4	31.8	4.1	1.2	0	23.3	15.0	3.8	35.3	17.3	2.3	2.3	0.8
Karnataka	39.2	6.1	6.9	17.7	21.1	8.4	0.2	0.3	54.1	28.1	6.3	0	3.0	7.8	0.1	0.5	43.7	16.1	9.7	9.1	11.0	9.0	0.7	0.7
Kerala	52.9	5.9	13.3	10.0	3.6	10.2	2.7	1.4	58.2	2.8	10.8	9.7	6.4	8.4	1.4	2.3	58.8	6.8	10.7	6.9	3.2	9.1	3.8	0.7
Maharashtra	40.2	15.9	17.0	10.8	1.9	11.7	2.3	0.3	41.4	19.2	15.4	8.3	2.6	10.6	2.2	0.3	47.1	21.5	12.6	5.8	2.0	8.7	1.7	0.6
Punjab	48.1	8.2	2.6	15.5	3.4	4.3	18.0	0	42.1	18.0	6.4	10.3	2.6	4.7	15.9	0	40.6	21.2	7.2	4.1	3.8	6.1	16.0	1.0
Tamil Nadu	31.3	9.5	20.3	21.1	12.2	3.7	1.4	0.5	38.0	19.4	19.2	14.8	3.2	3.4	1.0	1.0	38.1	21.9	19.7	13.4	3.4	2.5	0.6	0.5
Telangana	24.7	28.5	14.4	12.0	9.7	7.3	3.0	0.4	29.4	25.7	25.0	3.5	5.0	6.4	4.5	0.4	24.3	23.6	11.7	6.5	22.0	7.6	4.2	0.1
West Bengal	34.3	6.4	8.6	13.8	30.5	3.8	1.9	0.6	40.8	9.7	6.4	18.8	19.4	2.7	2.2	0.1	34.8	7.5	9.8	16.4	28.7	1.5	1.3	0.3

	2017								2018								2019								2020							
	**HG**	**IC**	**SI**	**CP**	**OTH**	**DR**	**JP**	**OP**	**HG**	**IC**	**SI**	**CP**	**OTH**	**DR**	**JP**	**OP**	**HG**	**IC**	**SI**	**CP**	**OTH**	**DR**	**JP**	**OP**	**HG**	**IC**	**SI**	**CP**	**OTH**	**DR**	**JP**	**OP**
**India**	45.6	17.3	10.8	11.3	6.7	4.9	2.6	0.8	47.4	16.7	9.0	10.9	6.9	5.2	3.0	0.8	49.2	17.3	7.9	9.9	7.5	5.4	2.2	0.6	52.1	18.0	6.0	9.1	6.2	6.0	1.9	0.8
Less developed states	45.7	13.9	11.3	16.2	6.5	3.3	2.2	0.9	49.4	13.3	9.6	14.3	6.5	3.7	2.3	0.9	50.7	15.2	8.9	12.5	6.0	4.1	1.8	0.9	54.7	15.0	6.9	11.8	3.8	4.9	1.8	1.0
Arunachal Pradesh	86.7	0	0	6.7	6.7	0	0	0	83.3	0	2.8	0	11.1	2.8	0	0	83.9	0	0	3.2	3.2	9.7	0	0	85.4	0	0	0	14.6	0	0	0
Assam	53.6	3.8	1.3	5.2	26.6	4.5	3.3	1.7	63.9	2.1	3.0	8.7	15.2	3.8	2.4	0.9	60.5	16.9	2.6	0	7.4	7.8	4.1	0.7	69.9	16.3	1.0	0	1.1	7.5	2.1	2.1
Bihar	46.9	13.0	2.1	3.1	11.5	15.1	0	8.3	47.6	15.7	3.2	2.7	6.5	8.1	8.1	8.1	52.8	14.2	7.3	2.0	8.1	6.9	4.5	4.1	60.3	11.5	5.3	3.6	3.6	8.4	1.4	5.9
Chhattisgarh	42.0	13.0	17.9	17.8	6.6	0.4	2.3	0.1	46.2	13.2	9.9	13.0	14.9	1.4	1.4	0.1	43.2	15.5	11.3	10.4	15.7	2.5	1.3	0	54.2	12.8	11.6	11.8	7.1	1.1	1.4	0
Jharkhand	41.1	5.8	6.7	1.8	3.1	17.2	12.0	12.3	49.3	9.1	9.3	0	0.2	15.9	2.5	13.7	56.2	16.0	6.6	1.6	1.6	6.9	1.4	9.8	49.4	19.9	4.7	4.6	1.4	12.1	1.6	6.2
Madhya Pradesh	46.6	24.5	14.7	7.6	1.2	4.0	1.1	0.2	47.9	25.7	14.1	5.8	2.1	3.2	0.9	0.3	52.6	23.9	10.7	5.7	2.5	2.9	1.2	0.6	54.0	22.0	9.9	8.1	0.5	4.5	0.7	0.3
Manipur	87.5	6.3	6.3	0	0	0	0	0	88.2	11.8	0	0	0	0	0	0	81.8	9.1	0	9.1	0	0	0	0	82.4	11.8	5.9	0	0	0	0	0
Meghalaya	75.0	0	2.8	8.3	2.8	5.6	5.6	0	89.5	0	0	2.6	7.9	0	0	0	90.9	3.0	0	0	3.0	3.0	0	0	95.1	0	0	1.6	1.6	0	1.6	0
Mizoram	93.3	6.7	0	0	0	0	0	0	91.7	0	0	0	8.3	0	0	0	100.0	0	0	0	0	0	0	0	95.0	0	5.0	0	0	0	0	0
Nagaland	100.0	0	0	0	0	0	0	0	100.0	0	0	0	0	0	0	0	100.0	0	0	0	0	0	0	0	100.0	0	0	0	0	0	0	0
Odisha	32.6	3.8	5.3	50.5	5.3	1.6	0.9	0.1	36.8	3.1	7.1	45.6	3.3	2.8	1.4	0	30.8	7.1	5.9	47.0	2.7	5.4	1.1	0.1	43.2	2.1	2.9	44.0	1.5	5.5	0.7	0
Rajasthan	48.9	10.7	5.2	8.8	11.0	7.1	7.9	0.4	55.7	8.4	2.8	10.5	2.6	8.0	11.7	0.3	59.2	8.0	4.0	9.1	5.5	8.6	5.0	0.5	58.4	11.8	1.7	7.5	4.5	8.5	6.5	1.2
Sikkim	96.2	0	0	0	3.8	0	0	0	96.4	0	0	0	0	1.8	1.8	0	98.3	0	0	0	0	0	1.7	0	88.2	1.5	0	0	8.8	0	1.5	0
Tripura	46.6	18.5	11.8	18.1	0	0.8	0.4	3.8	70.0	10.1	6.6	11.9	0	1.3	0	0	67.2	9.5	15.5	4.3	0	0.4	3.0	0	85.1	8.8	3.2	2.0	0	0.4	0.4	0
Uttar Pradesh	55.4	10.6	14.3	4.3	11.4	1.3	1.5	1.0	57.0	6.8	11.2	7.0	12.2	3.1	1.2	1.5	58.2	7.2	12.0	8.9	8.4	2.9	1.3	1.1	48.5	12.8	8.3	8.9	13.6	3.6	2.5	1.8
Uttarakhand	46.3	14.9	2.5	24.8	2.5	5.0	1.7	2.5	39.0	17.4	1.2	28.5	10.5	2.3	1.2	0	39.2	49.2	0.6	8.8	1.7	0.6	0	0	56.9	21.7	0	15.6	2.9	1.8	1.1	0
More developed states	45.5	19.0	10.7	8.9	6.6	5.7	2.7	0.8	46.2	18.4	8.7	9.3	7.1	6.0	3.4	0.8	48.4	18.6	7.5	8.5	8.1	6.0	2.4	0.5	50.8	19.6	5.6	7.8	7.0	6.6	1.9	0.6
Andhra Pradesh	35.6	27.6	9.9	9.4	6.7	5.7	4.4	0.7	40.5	27.6	3.4	14.3	1.7	7.7	3.9	0.8	40.2	29.9	3.5	9.6	4.8	8.1	3.6	0.5	47.9	24.7	2.9	15.8	1.3	4.2	2.2	1.1
Delhi	66.2	2.1	0.9	5.1	20.4	0.4	3.3	1.7	75.4	3.7	0.8	7.6	11.2	0.3	0.3	0.7	77.2	2.7	0.7	3.1	13.0	0.4	2.2	0.7	80.4	0.9	0.3	3.4	11.1	0.2	2.0	1.7
Goa	63.4	0	8.5	1.2	2.4	20.7	3.7	0	63.9	4.9	8.2	1.6	1.6	14.8	4.9	0	58.6	5.2	8.6	1.7	1.7	20.7	3.4	0	64.9	4.1	5.4	0	0	23.0	1.4	1.4
Gujarat	41.6	25.6	13.5	7.3	2.0	6.2	3.4	0.4	43.6	26.1	11.8	6.8	2.6	4.8	4.1	0.2	45.6	25.1	10.1	9.7	1.0	5.4	2.9	0.2	52.0	25.5	6.2	6.4	1.6	4.8	3.0	0.5
Haryana	31.7	5.9	3.7	19.6	22.9	4.4	11.2	0.6	33.9	6.8	1.7	18.6	20.4	5.2	12.9	0.5	31.1	10.6	2.2	14.4	29.8	4.1	7.5	0.2	42.9	8.9	1.2	17.1	19.6	4.9	5.3	0
Himachal Pradesh	29.2	58.3	6.7	0.4	3.3	2.1	0	0	33.6	53.8	1.6	2.8	5.3	2.4	0.4	0	46.9	48.8	2.4	0	0	1.9	0	0	44.3	48.0	2.0	1.0	0.3	1.7	2.4	0.3
Undivided Jammu & Kashmir	22.5	47.1	4.3	19.6	0.7	3.6	2.2	0	16.0	24.6	2.3	37.1	1.1	2.3	2.3	14.3	18.9	35.4	3.1	34.6	4.7	2.4	0.8	0	22.0	48.5	1.5	22.0	4.5	1.5	0	0
Karnataka	49.7	13.6	3.9	7.6	10.8	9.1	3.8	1.4	51.5	13.5	4.4	11.2	4.6	9.3	4.1	1.5	54.5	14.3	4.5	8.2	7.4	9.8	0.4	0.9	58.3	20.2	2.7	2.4	1.3	12.4	2.1	0.6
Kerala	62.6	7.9	9.4	3.2	3.8	9.0	2.6	1.6	64.9	5.7	10.5	5.0	3.3	7.8	2.0	0.9	64.1	6.7	11.9	3.5	2.4	8.4	2.3	0.6	65.0	6.0	11.2	3.6	3.2	9.1	1.2	0.7
Maharashtra	47.6	21.7	11.1	3.7	3.2	9.0	3.0	0.7	51.7	21.5	8.3	4.5	0.9	10.0	2.6	0.6	53.9	23.0	6.5	3.2	1.4	8.9	2.4	0.7	56.9	22.0	4.2	2.9	1.4	9.8	2.5	0.3
Punjab	54.4	11.6	2.4	10.1	0.6	7.3	12.2	1.2	56.4	18.7	1.4	7.0	0.8	3.6	11.7	0.3	48.1	18.9	2.6	5.8	5.4	5.2	12.1	2.0	58.7	20.5	2.7	6.1	2.4	4.3	4.3	0.8
Tamil Nadu	41.9	21.5	17.9	12.1	1.9	2.9	0.9	1.0	41.0	22.8	15.6	9.2	2.9	5.0	2.8	0.7	49.0	18.9	14.8	9.5	0.1	5.1	2.2	0.4	45.2	24.5	10.8	10.1	0.7	6.6	0.9	1.3
Telangana	38.5	33.6	11.5	4.1	2.3	6.1	3.7	0.2	37.4	33.5	10.6	0.5	5.7	4.7	7.3	0.3	30.6	38.1	8.7	6.1	6.6	5.2	4.4	0.3	32.0	44.8	4.5	4.7	0.2	9.1	3.9	0.8
West Bengal	46.2	11.3	10.4	16.0	13.0	2.4	0.3	0.5	41.7	8.8	7.5	15.7	21.3	2.7	1.4	0.8	44.3	8.3	4.6	14.4	25.3	2.6	0.3	0.2	46.2	4.5	4.6	13.3	29.2	2.0	0.1	0.1
